# Preparation, characterization and catalytic application of nano-Fe_3_O_4_@SiO_2_@(CH_2_)_3_OCO_2_Na as a novel basic magnetic nanocatalyst for the synthesis of new pyranocoumarin derivatives†

**DOI:** 10.1039/c8ra05501g

**Published:** 2018-08-03

**Authors:** Hamideh Mohamadi Tanuraghaj, Mahnaz Farahi

**Affiliations:** Department of Chemistry, Yasouj University Yasouj Iran 75918-74831 farahimb@yu.ac.ir (+98)7412242167

## Abstract

We present a study on the synthesis, characterization and application of sodium carbonate tag silica-coated nano-Fe_3_O_4_ (Fe_3_O_4_@SiO_2_@(CH_2_)_3_OCO_2_Na) as a novel and efficient heterogeneous basic catalyst. The described catalyst was fully characterized *via* FT-IR, X-ray diffraction (XRD), energy dispersive X-ray spectroscopy (EDS), and field emission scanning electron microscopy (FE-SEM). The reported novel magnetic nanocatalyst presents an excellent activity and catalytic performance for the synthesis of a novel series of pyranocoumarins through the reaction of dialkyl acetylenedicarboxylates and 5,7-dihydroxy coumarin derivatives at 100 °C under solvent-free conditions.

## Abstract 2

We present a study on the synthesis, characterization and application of sodium carbonate tag silica-coated nano-Fe_3_O_4_ (Fe_3_O_4_@SiO_2_@(CH_2_)_3_OCO_2_Na) as a novel and efficient heterogeneous basic catalyst.

## Graphical abstract

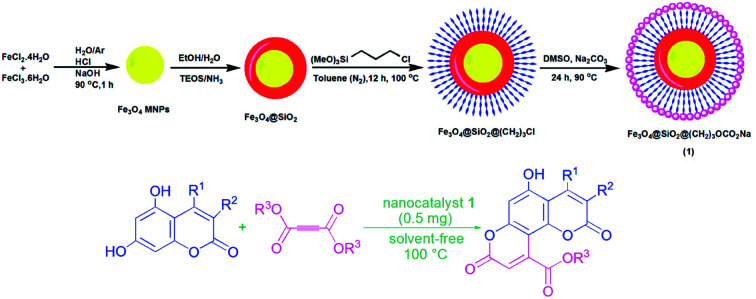

## Introduction

In recent years, there has been a pronounced tendency to utilize heterogeneous catalysts.^1^ The reason why heterogeneous catalysts are attractive is their unique properties including easy separation, low toxicity, air tolerance, easy handling, and reusability.^2–4^ Fe_3_O_4_ magnetic nanoparticles (MNPs) have been widely used in design of environmentally benign heterogeneous catalysts.^5^ The reason for the above choice is their large surface areas, good textural properties, supermagnetism, high coercivity and low Curie temperature as well as non-toxicity.^6–8^ A striking feature of magnetic nanocatalysts is that they can be readily separated using an external magnet, which achieves a simple separation of the catalyst without filtration.^9^ Also, they possess high potential active sites for loading of other functional groups.^10^ To prevent Fe_3_O_4_ nanoparticles from undergoing oxidation in an air atmosphere and in order to increase the surface area and simplify the surface functionalization, a protective shell of silica can be formed onto their surface.^11,12^

Pyranocoumarins with excellent chemical and physical characteristics have acquired substantial attention and show great practical values in many fields, such as medicine discovery, dye chemistry, and materials chemistry. Structural diversity associated with pyranocoumarins has resulted in a large number of new molecular entities, which have been found to be useful as anti-cancer, anti-oxidant, anti-inflammatory, anti-allergic, hepatoprotective, anti-viral, anti-carcinogenic agents, enzyme inhibitor, and precursor of toxic substances.^13–16^ In view of the biological, industrial, and synthetic importance of pyranocoumarins, several methods for their synthesis have been reported.^17–19^ Although most of these processes have distinct advantages, the use of high temperatures, environmentally harmful catalysts, harsh reaction conditions, long reaction times and large quantity of volatile organic solvents limit the use of these methods. Therefore, the search for the possibility synthesis of these compounds under mild reaction conditions with recoverable effective heterogeneous catalyst is still in high demand.

In continuation of our efforts to design, synthesis and application of novel nanocatalyst,^20,21^ we have immobilized sodium carbonate on silica-coated Fe_3_O_4_ magnetic nanoparticles (Fe_3_O_4_@SiO_2_@(CH_2_)_3_OCO_2_Na) and then investigated its performance as novel strong, recoverable, and stable basic nanocatalyst for synthesis of new pyranocoumarin derivatives.

## Experimental

All chemical reagents were purchased from Merck and Sigma Aldrich companies and used without further purification. Melting points were determined in open capillaries using an electrothermal KSB1N-apparatus (Krüss, Germany). FT-IR spectra were obtained with potassium bromide pellets in the range 400–4000 cm^−1^ with a FT-IR-680 plus spectrometer (JASCO, Japan). ^1^H NMR and ^13^C NMR spectra were recorded on a FT-NMR Bruker Avance Ultra Shield Spectrometer (Bruker, USA) at 400 and 100 MHz, respectively. X-ray diffraction pattern of the prepared catalyst was obtained using D_8_ ADVANCE X-ray diffraction using Co-Kα radiation (*λ* = 1.7890 Å) (Bruker, Germany). Energy dispersive spectroscopy (EDS) was performed using TESCAN Vega model instrument. The morphology of the particles was studied by Field Emission Scanning Electron Microscopy (FE-SEM) in a MIRA3TESCAN-XMU FE-SEM instrument.

### Synthesis of Fe_3_O_4_ MNPs

A mixture of FeCl_3_·6H_2_O (2.3 g, 8.7 mmol) and FeCl_2_·4H_2_O (0.86 g, 4.3 mmol) was dissolved in deionized water (100 mL). The solution was heated to 90 °C under nitrogen atmosphere and stirred about 30 min. Subsequently, sodium hydroxide solution (10 mL, 25%) was added dropwise to the solution until the brown color solution turned out to the black. After approximately 1 h, the black precipitate isolated in a magnetic field from the reaction mixture, repeatedly washed with deionized water several times to remove the remaining impurities.^22^

### Preparation of Fe_3_O_4_@SiO_2_

Dried Fe_3_O_4_ nanoparticles (0.5 g) was suspended in a mixture of ethanol (20 mL) and NH_3_·H_2_O (5 mL, 25%) followed by the addition of tetraethoxysilane (TEOS) (0.3 g) to the solution and the mixture was ultrasonicated for 2 h. Next, the mixture was degassed and stirred for 24 h.^23^

### Procedure for the synthesis of Fe_3_O_4_@SiO_2_@(CH_2_)_3_Cl

The prepared Fe_3_O_4_@SiO_2_ (0.5 g) was dispersed in dry toluene (80 mL) by sonication for 15 min, then, 3-chloropropyltriethoxysilan (0.121 g, 0.5 mmol) was added to the mixture and heated to 100 °C under reflux condition and N_2_ atmosphere. After 12 h, Fe_3_O_4_@SiO_2_@(CH_2_)_3_Cl was separated magnetically and washed with deionized water and ethanol.^24^

### Preparation of Fe_3_O_4_@SiO_2_@(CH_2_)_3_OCO_2_Na

Fe_3_O_4_@SiO_2_@(CH_2_)_3_Cl (0.5 g) was dissolved in DMSO (50 mL) by sonication. Then Na_2_CO_3_ (1 g) was added to the above mixture and heated to the 90 °C under reflux condition for 24 h. Next, the obtained black precipitate was separated by a normal magnet an washed with distilled water and ethanol and dried overnight under vacuum at 60 °C.

### General procedure for the synthesis of pyranocoumarins 4

In a round bottom flask, a mixture of 5,7-dihydroxy coumarins 2 (1 mmol), dialkyl acetylene dicarboxylate 3 (1.5 mmol) and prepared magnetic nanocatalyst 1 (0.0005 g) was heated to 100 °C. The progress of the reaction was monitored by TLC (*n*-hexane : EtOAc/3 : 1 (v/v)). After completion of the reaction which was distinguished by disappearing of starting materials' spots on TLC, the reaction mixture was diluted by methanol and the catalyst was easily separated magnetically using an external magnet. The desired product was purified using column chromatography (using ethylacetate : *n*-hexane, 3 : 1 as mobile phase).

### General procedure for the recovery of nanocatalyst 1

5,7-Dihydroxy-4-methyl coumarin (1 mmol), dimethyl acetylenedicarboxylates (1.5 mmol), and Fe_3_O_4_@SiO_2_@(CH_2_)_3_OCO_2_Na (0.0005 g) were stirred at free-solvent condition in 100 °C for 6 h. After completion of the reaction, methanol (5 mL) was added to the reaction mixture and the catalyst was separated by an external magnet. It was washed three times by EtOH (10 mL) and deionized water (10 mL) and then dried under vacuum at 80 °C.

#### Methyl 5-hydroxy-4-methyl-2,8-dioxo-2*H*,8*H*-pyrano[2,3-*f*]chromene-10-carboxylate (4a)

Yellow crystals (0.278 g), IR (KBr): *ν*_max_ = 3444, 2935, 1739, 1675, 1623, 1438, 1235, 623 cm^−1^. ^1^H NMR (400 MHz, DMSO-*d*_6_): *δ* = 10.40 (s, 1H), 6.65 (s, 1H), 6.40 (s, 1H), 5.97 (s, 1H), 3.51 (s, 3H), 1.81 (s, 3H). ^13^C NMR (100 MHz, DMSO-*d*_6_): *δ* = 167.8, 161.6, 160.1, 158.9, 156.0, 152.9, 151.1, 147.1, 120.7, 118.7, 113.2, 111.2, 103.0, 50.4, 27.8. Anal. calcd for C_15_H_10_O_7_: C, 59.61; H, 3.34. Found: C, 59.69; H, 3.29.

#### Methyl 4-(chloromethyl)-5-hydroxy-2,8-dioxo-2*H*,8*H*-pyrano[2,3-*f*]chromene-10-carboxylate (4b)

Creamy crystals (0.285 g), IR (KBr): *ν*_max_ = 3424, 2956, 1723, 1660, 1617, 1434, 1278, 649 cm^−1^. ^1^H NMR (400 MHz, DMSO-*d*_6_): *δ* = 10.75 (s, 1H), 6.76 (s, 1H), 6.25 (s, 1H), 6.08 (s, 1H), 4.21 (s, 2H), 3.38 (s, 3H). ^13^C NMR (100 MHz, DMSO-*d*_6_): *δ* = 167.8, 161.8, 160.8, 160.1, 157.6, 154.5, 152.8, 144.5, 121.5, 118.7, 114.0, 111.7, 106.8, 50.4, 49.0. Anal. calcd for C_15_H_9_ClO_7_: C, 53.51; H, 2.69. Found: C, 53.54; H, 2.67.

#### Ethyl 5-hydroxy-4-methyl-2,8-dioxo-2*H*,8*H*-pyrano[2,3-*f*]chromene-10-carboxylate (4c)

Pale yellow crystals (0.274 g), IR (KBr): *ν*_max_ = 3439, 2972, 1738, 1658, 1622, 1439, 1281, 646 cm^−1^. ^1^H NMR (400 MHz, DMSO-*d*_6_): *δ* = 10.48 (s, 1H), 6.87 (s, 1H), 6.57 (s, 1H), 6.10 (s, 1H), 6.07 (q, 2H, *J* = 8 Hz), 2.10 (s, 3H), 1.21 (t, 3H, *J* = 4 Hz). ^13^C NMR (100 MHz, DMSO-*d*_6_): *δ* = 168.8, 161.9, 160.8, 160.1, 160.0, 155.5, 150.1, 147.1, 140.7, 119.0, 116.0, 112.0, 109.2, 102.6, 62.2, 24.2, 14.1. Anal. calcd for C_16_H_12_O_7_: C, 60.76; H, 3.82. Found: C, 60.71; H, 3.90.

#### Ethyl 4-(chloromethyl)-5-hydroxy-2,8-dioxo-2*H*,8*H*-pyrano[2,3-*f*]chromene-10-carboxylate (4d)

Yellow crystals (0.280 g), IR (KBr): *ν*_max_ = 3440, 2963, 1748, 1661, 1633, 1445, 1279, 639 cm^−1^. ^1^H NMR (400 MHz, DMSO-*d*_6_): *δ* = 10.76 (s, 1H), 6.63 (s, 1H), 6.33 (s, 1H), 6.25 (s, 1H), 4.80 (s, 1H), 4.31 (q, 2H, *J* = 7 Hz), 1.32 (t, 3H, *J* = 4 Hz). ^13^C NMR (100 MHz, DMSO-*d*_6_): *δ* = 169.4, 163.4, 160.6, 157.8, 157.2, 152.8, 151.4, 147.7, 119.3, 117.4, 114.7, 110.7, 105.8, 62.4, 56.2, 15.0. Anal. calcd for C_16_H_11_ClO_7_: C, 54.80; H, 3.16. Found: C, 54.78; H, 3.20.

#### Ethyl 11-hydroxy-2,6-dioxo-7,8,9,10-tetrahydro-2*H*,6*H*-benzo[*c*]pyrano[2,3-*h*]chromene-4-carboxylate (4e)

White crystals (0.256 g), IR (KBr): *ν*_max_ = 3437, 2977, 1743, 1665, 1641, 1458, 1277, 650 cm^−1^. ^1^H NMR (400 MHz, DMSO-*d*_6_): *δ* = 10.50 (s, 1H), 6.63 (s, 1H), 6.16 (s, 1H), 4.07 (q, 2H, *J* = 8 Hz), 1.22 (t, 3H, *J* = 4 Hz). ^13^CNMR (100 MHz, DMSO-*d*_6_): *δ* = 168.3, 162.5, 159.8, 155.9, 152.3, 150.6, 149.6, 147.3, 122.0, 118.3, 116.1, 111.1, 103.7, 61.6, 28.6, 28.2, 22.0, 21.6, 14.1. Anal. calcd for C_19_H_16_O_7_: C, 64.04; H, 4.53. Found: C, 64.10; H, 4.55.

#### Methyl 11-hydroxy-2,6-dioxo-7,8,9,10-tetrahydro-2*H*,6*H*-benzo[*c*]pyrano[2,3-*h*]chromene-4-carboxylate (4f)

Creamy crystals (0.249 g), IR (KBr): *ν*_max_ = 3440, 2975, 1738, 1668, 1646, 1462, 1281, 653 cm^−1^. ^1^H NMR (400 MHz, DMSO-*d*_6_): *δ* = 10.49 (s, 1H), 6.94 (s, 1H), 6.21 (s, 1H), 4.22 (s, 1H), 1.11–0.98 (m, 8H). ^13^C NMR (100 MHz, DMSO-*d*_6_): *δ* = 165.0, 161.7, 159.4, 159.1, 153.1, 151.7, 147.9, 147.5, 118.3, 117.9, 116.8, 113.7, 99.9, 52.5, 29.5, 24.7, 23.3, 22.3. Anal. calcd for C_19_H_16_O_7_: C, 64.04; H, 4.53. Found: C, 64.09; H, 4.60.

#### Methyl 5-hydroxy-2,8-dioxo-4-phenyl-2*H*,8*H*-pyrano[2,3-*f*]chromene-10-carboxylate (4g)

Yellow crystals (0.327 g), IR (KBr): *ν*_max_ = 3438, 2971, 1742, 1663, 1649, 1457, 1280, 649 cm^−1^. ^1^H NMR (400 MHz, DMSO-*d*_6_): *δ* = 10.45 (s, 1H), 7.28–7.11 (m, 3H), 7.12 (d, 2H, *J* = 8 Hz), 6.63 (s, 1H), 6.59 (s, 1H), 6.00 (s, 1H), 3.70 (s, 3H). ^13^C NMR (100 MHz, DMSO-*d*_6_): *δ* = 170.2, 159.4, 158.9, 153.8, 152.5, 152.3, 146.5, 142.6, 129.3, 128.3, 127.1, 118.9, 117.7, 113.0, 110.4, 103.6, 52.7. Anal. calcd for C_20_H_12_O_7_: C, 65.94; H, 3.32. Found: C, 65.89; H, 3.36.

## Results and discussion

**Scheme 1:**
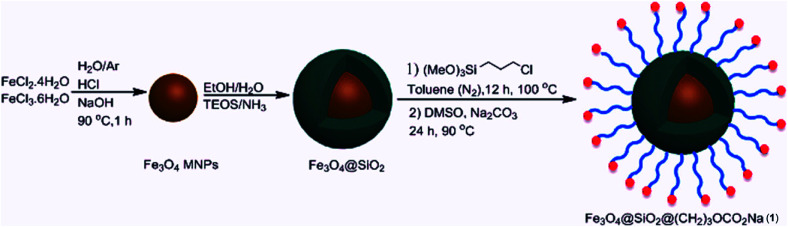
Synthesis of Fe_3_O_4_@SiO_2_@(CH_2_)_3_OCO_2_Na (1).

**Fig. 1:**
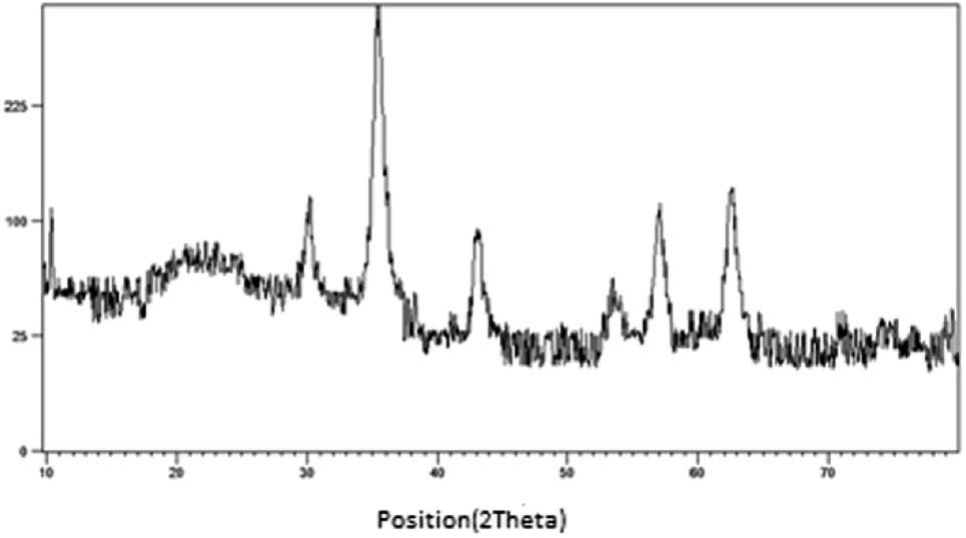
The XRD patterns of Fe_3_O_4_@SiO_2_@(CH_2_)_3_OCO_2_Na.

Due to reasonable needs to clean and green heterogeneous basic catalysts, the magnetic nanocatalyst Fe_3_O_4_@SiO_2_@(CH_2_)_3_OCO_2_Na (1) was synthesized following the procedure shown in Scheme 1. The structure of prepared nanocatalyst 1 was studied and fully characterized using FT-IR, EDS, XRD, and FE-SEM analysis. These results provided the evidences that the expected structure was successfully achieved. Fig. 1 presents the X-ray diffraction (XRD) patterns of the magnetic nanocatalyst 1. As can be seen, six characteristic peaks over 2*θ* range from 10 to 80° at 30.86, 35.36, 43.96, 54.36, 57.26, and 62.56 corresponding to the (220), (311), (400), (422), (511) and (440) crystal planes indicate the cubic spinal structure of Fe_3_O_4_ MNPs.^25^ The characteristic peaks confirming the presence of –OCO_2_Na is appeared around 10, 34, 36 and 43 (2*θ*) which some of them is overlapped by the Fe_3_O_4_ peaks. However, the appearance of a peak at 10.53 confirms the connection of –OCO_2_Na in nanocatalyst.^26^ Additionally, a broad peak at 13–28 in XRD pattern of catalyst indicates the existence of amorphous silica in the structure of the catalyst.^27^

**Fig. 2:**
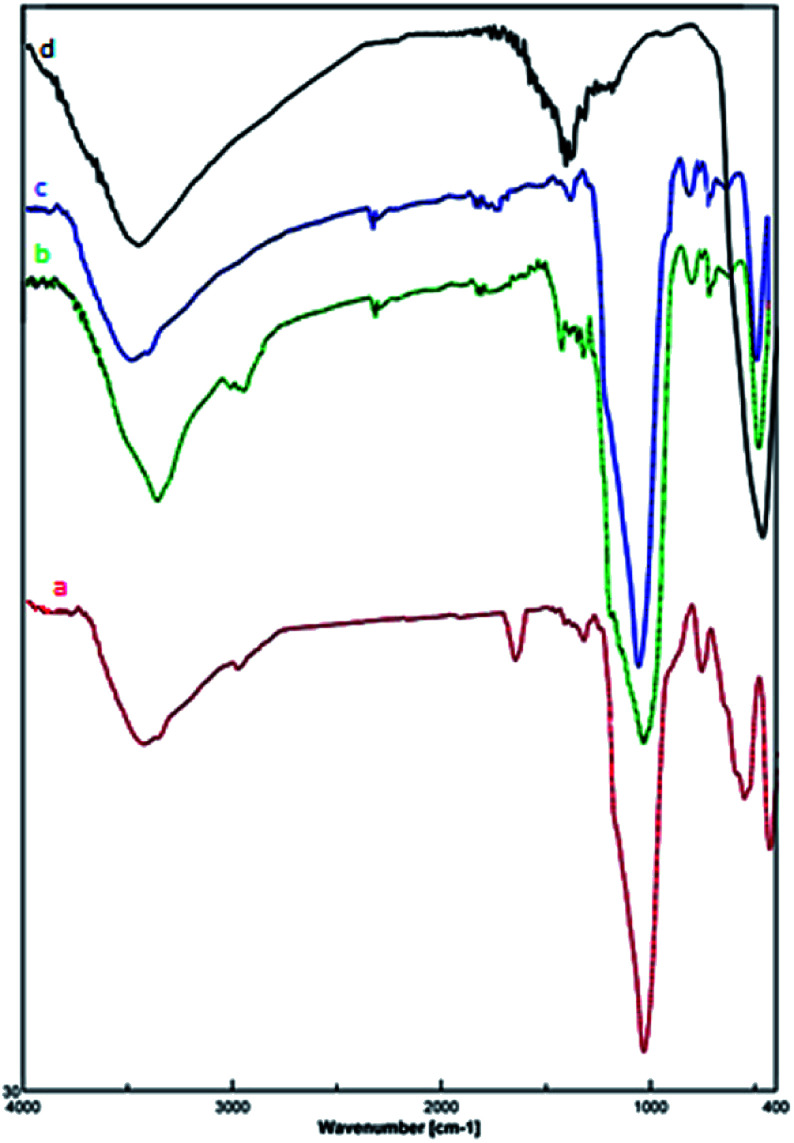
FT-IR spectra of (a) Fe_3_O_4_@SiO_2_@(CH_2_)_3_OCO_2_Na, (b) Fe_3_O_4_@SiO_2_(CH_2_)_3_Cl, (c) Fe_3_O_4_@SiO_2_, and (d) Fe_3_O_4_ MNPs.

The structure of prepared nanocatalyst 1 was confirmed by the FT-IR spectra (Fig. 2). The sample exhibits a peak at 632 cm^−1^ band that is due to the stretching vibration mode associated to the Fe–O absorption band. The stretch found at 466 cm^−1^ is related to the presence of Fe–O–Si bond in the sample.^28^ Furthermore, the spectra present the O–H stretching vibrational around 3431 cm^−1^ and two absorption peaks at 798 cm^−1^ and 1084 cm^−1^ which corresponds to the symmetric and asymmetric stretching vibration of Si–O.^29^ The band at 2928 cm^−1^ is attributed to the alkyl chain –CH_2_.^30^ The presence of the –OCO_2_Na group is confirmed by the bands at 1636 cm^−1^ and 1407 cm^−1^.^26^

**Fig. 3:**
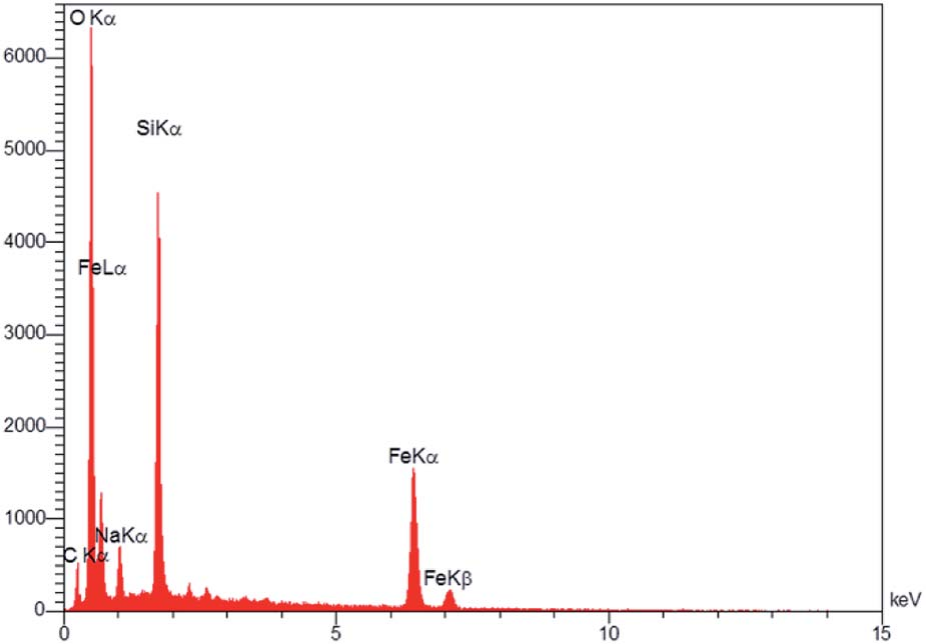
EDS analysis of Fe_3_O_4_@SiO_2_@(CH_2_)_3_OCO_2_Na.

The prepared magnetic nanocatalyst was analyzed by using an energy dispersive spectrometer (EDS). According to the Fig. 3, it is seen that Fe_3_O_4_@SiO_2_@(CH_2_)_3_OCO_2_Na contains all expected elemental cases including Si, O, Fe, C and Na.

**Fig. 4:**
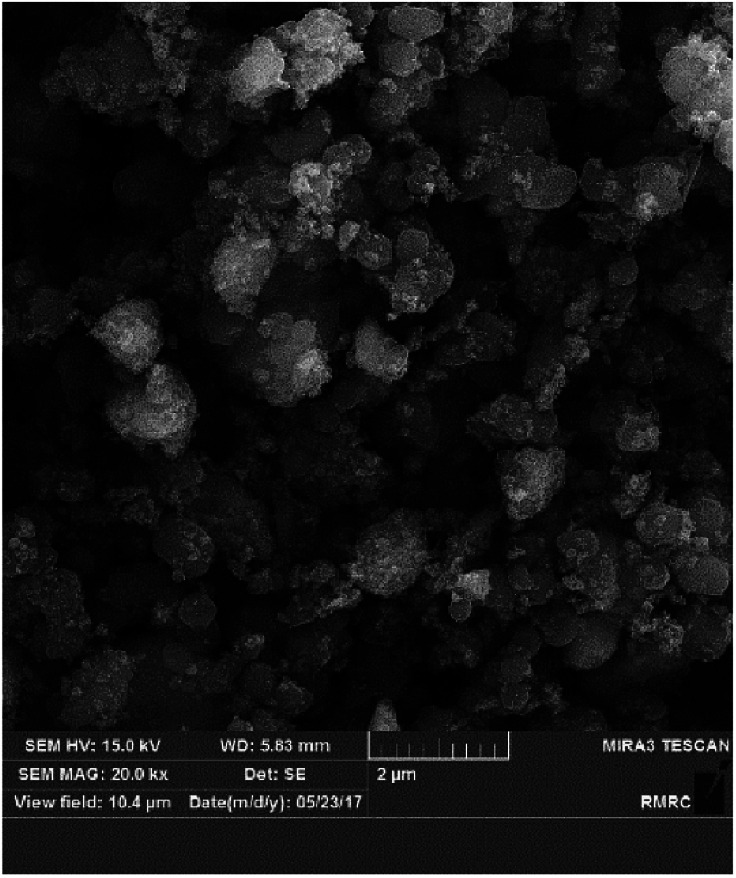
FE-SEM analysis of nanocatalyst 1.

The FE-SEM image of Fe_3_O_4_@SiO_2_@(CH_2_)_3_OCO_2_Na was shown in Fig. 4. The image demonstrates uniform-size particles with near spherical morphology. As it comes from FE-SEM analysis the average diameter of obtained nanoparticles is around 25 nm.

**Fig. 5:**
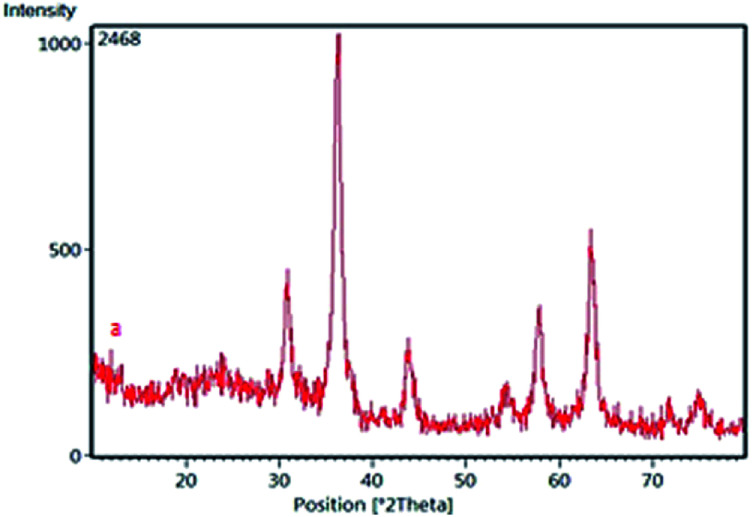
The XRD patterns of recycled catalyst.

To test the stability of the catalyst structure, the recycled nano catalyst was examined by XRD analysis; the diffraction patterns and relative intensities of all peaks matched well with those of the primary catalyst (Fig. 5).

**Scheme 2:**
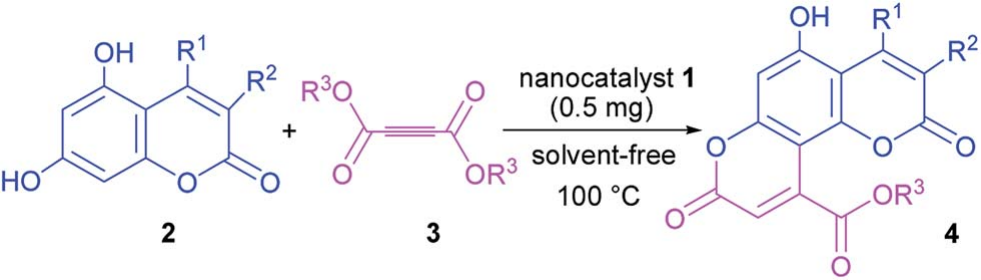
Fe_3_O_4_@SiO_2_@(CH_2_)_3_OCO_2_Na-catalyzed synthesis of pyranocoumarins 4.

Having successfully prepared the new nanocatalyst 1, further studies were performed for the synthesis of a novel class of pyranocoumarins 4*via* the condensation of 5,7-dihydroxycoumarins 2 and dialkyl acetylenedicarboxylates 3 (Scheme 2). The structure of product was confirmed by FT-IR, ^1^H NMR, ^13^C NMR, and elemental analysis. Initially, 5,7-dihydroxycoumarins (2) was synthesized according to the reported procedure.^31^

In order to obtain the optimal experimental conditions, we set up a model reaction between 5,7-dihydroxy-4-methyl coumarin and dimethyl acetylenedicarboxylate (DMAD). The model reaction was conducted in the presence of several homogeneous and heterogeneous catalysts at various conditions. It was observed that the best result was achieved using 0.5 mg nanocatalyst 1 at 100 °C under solvent-free conditions. The results are summarized in Table 1.

**Table 1:** Screening for the model reaction

Entry	Solvent	Catalyst	*T* (°C)	Time (h)	Yield^a^ (%)
1	—	—	25	24	5
2	Toluene	—	25	24	8
3	Toluene	—	Reflux	12	12
4	Toluene	NaOH (5 mol%)	Reflux	12	25
5	Toluene	Na_2_CO_3_ (5 mol%)	Reflux	12	20
6	CHCl_3_	NaOH (5 mol%)	Reflux	12	15
7	CHCl_3_	Na_2_CO_3_ (5 mol%)	Reflux	12	15
8	CH_2_Cl_2_	Na_2_CO_3_ (5 mol%)	Reflux	12	14
9	MeOH	NaOH (5 mol%)	Reflux	12	10
10	MeOH	Na_2_CO_3_ (5 mol%)	Reflux	12	12
11	EtOAc	NaOH (5 mol%)	Reflux	12	25
12	EtOAc	Na_2_CO_3_ (5 mol%)	Reflux	12	29
13	EtOAc	Catalyst 1 (0.1 mg)	Reflux	6	70
14	EtOAc	Catalyst 1 (0.3 mg)	Reflux	6	80
15	EtOAc	Catalyst 1 (0.5 mg)	Reflux	6	92
16	EtOAc	Catalyst 1 (0.7 mg)	Reflux	6	90
17	Toluene	Catalyst 1 (0.5 mg)	Reflux	6	70
18	CH_2_Cl_2_	Catalyst 1 (0.5 mg)	Reflux	6	75
19	CHCl_3_	Catalyst 1 (0.5 mg)	Reflux	6	80

a Isolated yield.

After establishing the optimal conditions, the efficiency of the catalyst was further evaluated with a series of 5,7-dihydroxy coumarins. Furthermore, the procedure worked well for diethyl acetylenedicarboxylate as well as DMAD (Table 2).

**Table 2:** Preparation of pyranocoumarins 4 in the presence of Fe_3_O_4_@SiO_2_@(CH_2_)_3_OCO_2_Na^a^

Entry	Product^b^	Time (h)	Yield^c^ (%)	Mp (°C)
4a	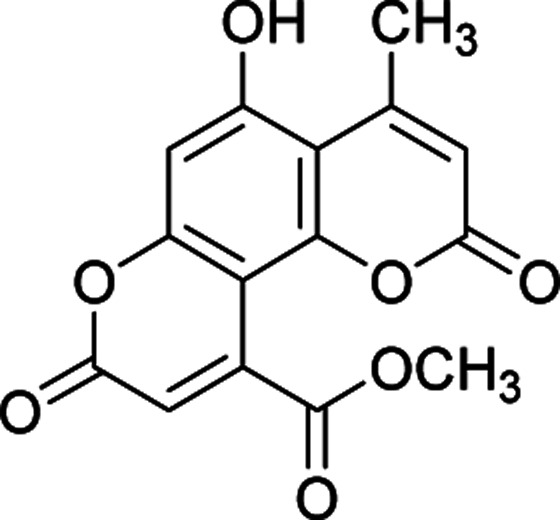	6	92	243–244
4b	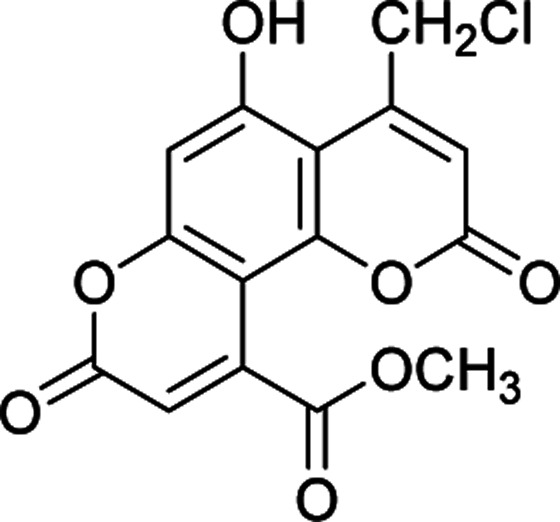	7	85	278–279
4c	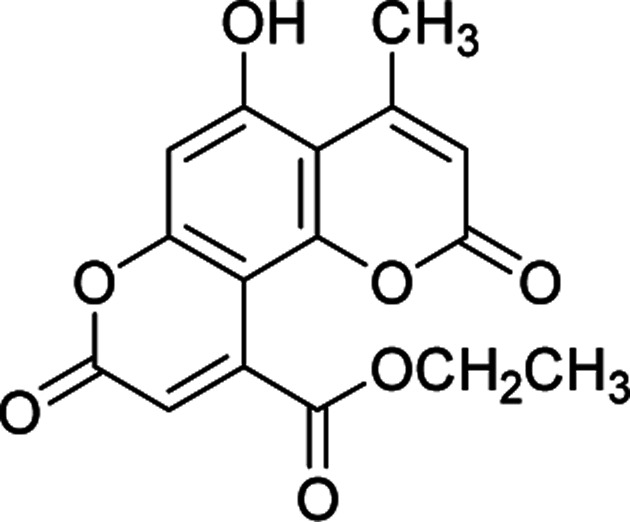	5	87	261–263
4d	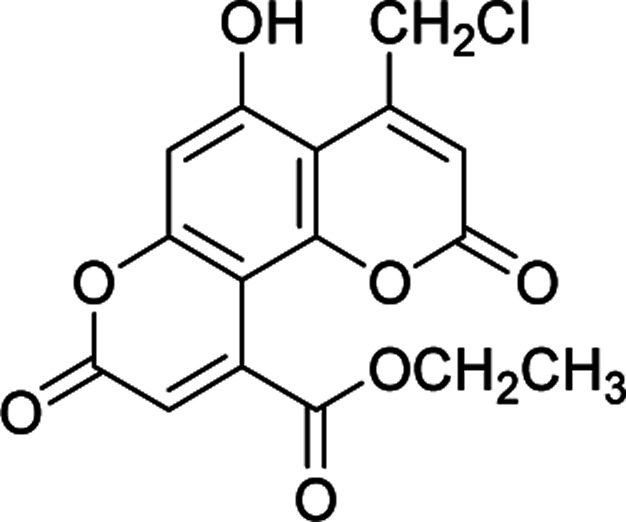	7	80	272–273
4e	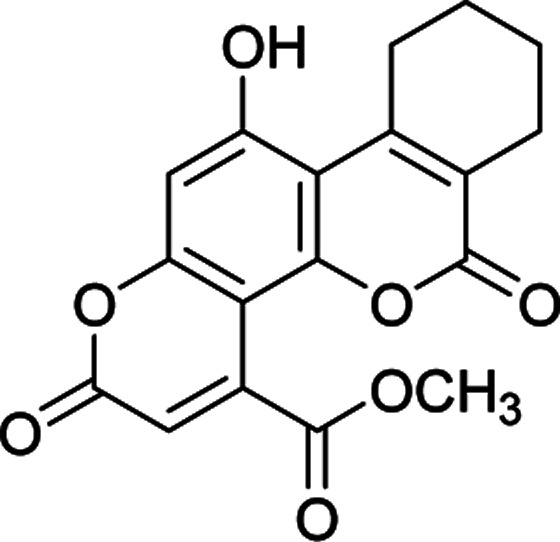	8	75	237–239
4f	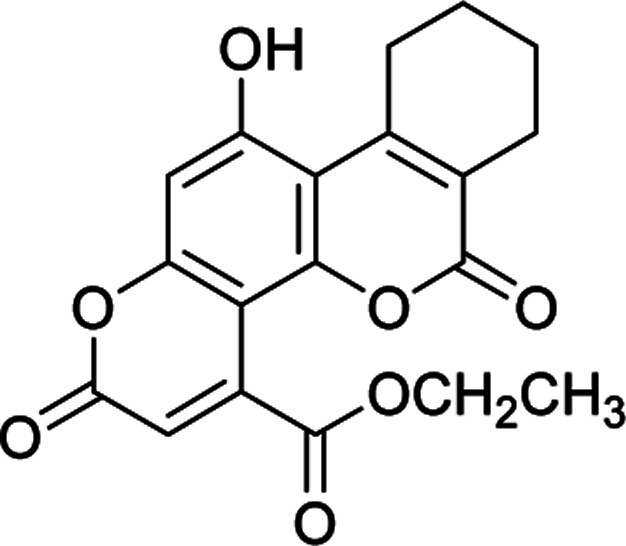	6	70	269–270
4g	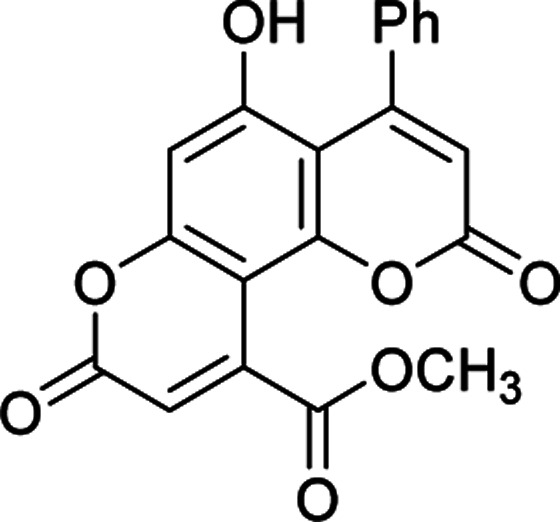	9	90	277–278

a Reaction conditions: 5,7-dihydroxycoumarins 2 (1 mmol), dialkyl acetylenedicarboxylates 3 (1.5 mmol) and nanocatalyst 1 (0.0005 g) at 100 °C.

b All products are novel and characterized by IR, ^1^H NMR, ^13^C NMR, and elemental analysis.

c Isolated yield.

**Scheme 3:**
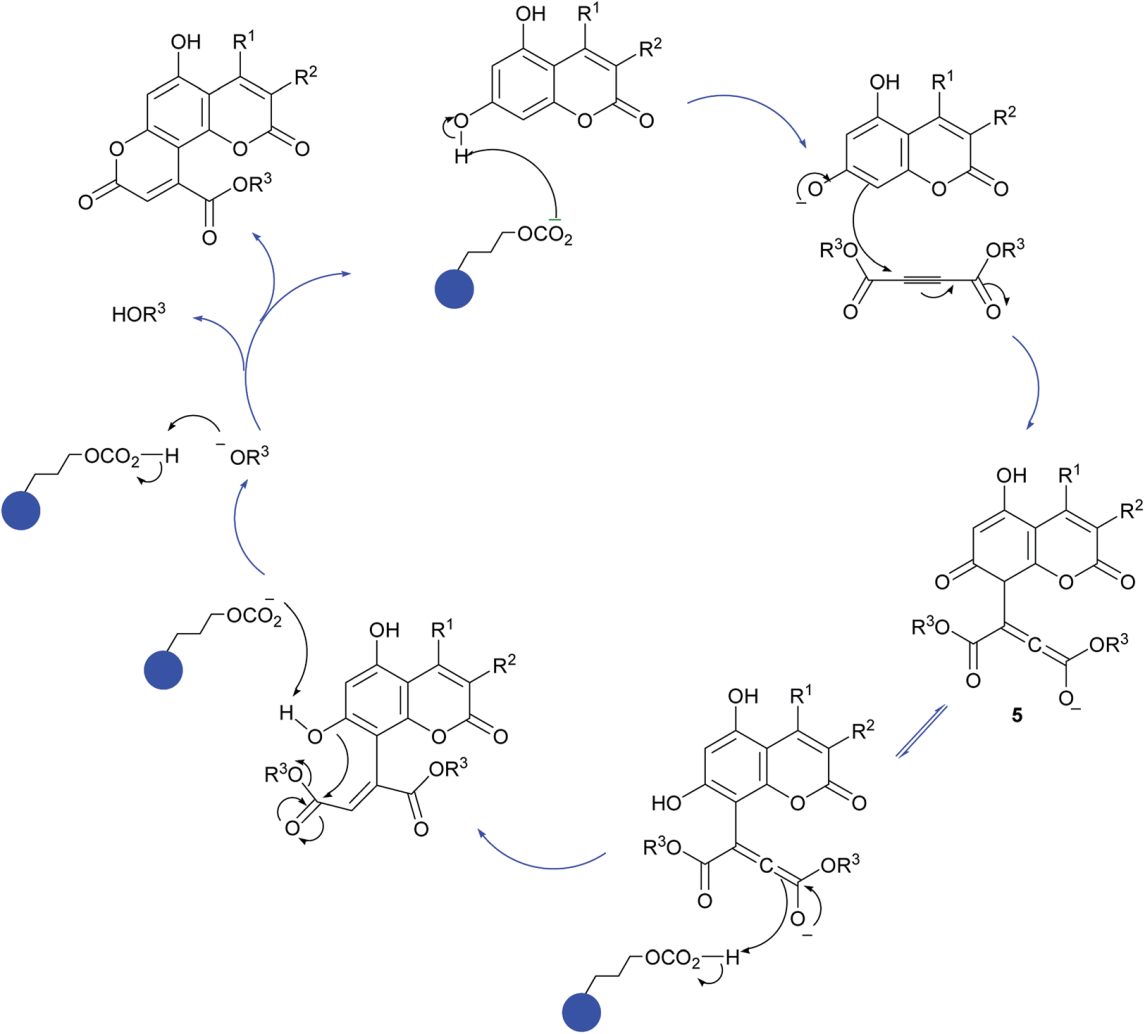
Proposed mechanism for the synthesis of pyranocoumarin 4.

A plausible mechanism for the formation of pyranocoumarin derivatives catalyzed by Fe_3_O_4_@SiO_2_@(CH_2_)_3_OCO_2_Na is shown in Scheme 3. According to the reaction pathway, in the beginning, the OH group on the C-7 of coumarin attacks the dialkyl acetylenedicarboxylate to give intermediate 5. The role of catalyst comes in this stage where it removes the hydrogen of OH group and facilitates the attack of coumarin to the dialkyl acetylenedicarboxylate. Unstable intermediate 5 regains the previous stable aromatic form *via* keto–enol equilibrium. Then the dehydrogenated oxygen of position 7 attack the carbonyl group of dialkyl acetylenedicarboxylate portion and by removing of the HOR^3^ forms the desired product.

**Fig. 6:**
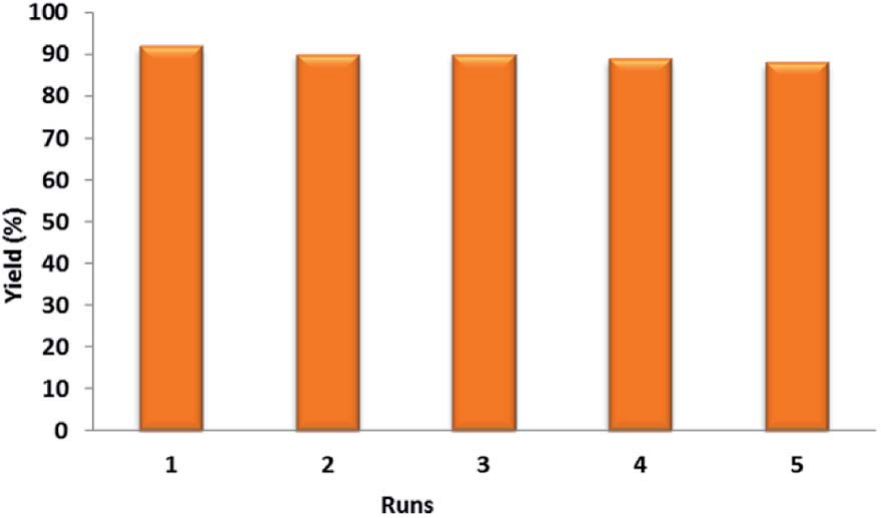
Reusability study of nanocatalyst 1 in the synthesis of 4a at 100 °C under solvent-free conditions.

To examine the reusability of the prepared nanocatalyst, after completion of the model reaction, the reaction mixture was diluted with methanol and the catalyst was magnetically removed from it. Afterwards, the isolated catalyst was washed by ethanol and deionized water and dried. The separated catalyst was directly used for the next run under the same condition. The results showed that the catalyst can be reused, without deactivation, even after five cycles (Fig. 6).

## Conclusions

In summary, we have reported Fe_3_O_4_@SiO_2_@(CH_2_)_3_OCO_2_Na as a new and efficient basic nanocatalyst. It was fully characterized by FT-IR, FE-SEM, EDS and XRD analysis. Furthermore, herein, the first application of Fe_3_O_4_@SiO_2_@(CH_2_)_3_OCO_2_Na as a green, convenient and recyclable catalyst to the synthesis of new pyranocoumarins is successfully examined. The introduced reaction reveals broad substrate scope, good functional group tolerance, solvent-free condition and good to excellent yields. The structure of products was confirmed by IR, ^1^H NMR, ^13^C NMR, and elemental analysis.

## Conflicts of interest

There are no conflicts to declare.

## Supplementary Material

RA-008-C8RA05501G-s001
