# Supplementary material for: Preparation, characterization and catalytic application of nano-Fe3O4@SiO2@(CH2)3OCO2Na as a novel basic magnetic nanocatalyst for the synthesis of new pyranocoumarin derivatives
Source: RSC Adv. 2018 Aug 3;8(49):27818–24. doi: 10.1039/c8ra05501g (PMC9083525; doi:10.1039/c8ra05501g)
Supplement: RA-008-C8RA05501G-s001 [file RA-008-C8RA05501G-s001.pdf]

Electronic Supplementary Material (ESI) for RSC Advances.
This journal is © The Royal Society of Chemistry 2018

Supporting Information

Preparation, characterization and catalytic application of nano-Fe_3O_4@SiO_2@$(CH_2)_3OCO_2Na$ as a novel basic magnetic nanocatalyst for the synthesis of new pyranocoumarin derivatives

Hamideh Mohamadi Tanuraghaj and Mahnaz Farahi*

Department of Chemistry, Yasouj University, Yasouj, Iran, Zip Code: 75918-74831, Fax: (+98)7412242167. E-mail: farahimb@yu.ac.ir

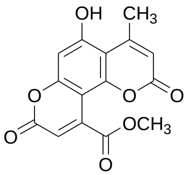

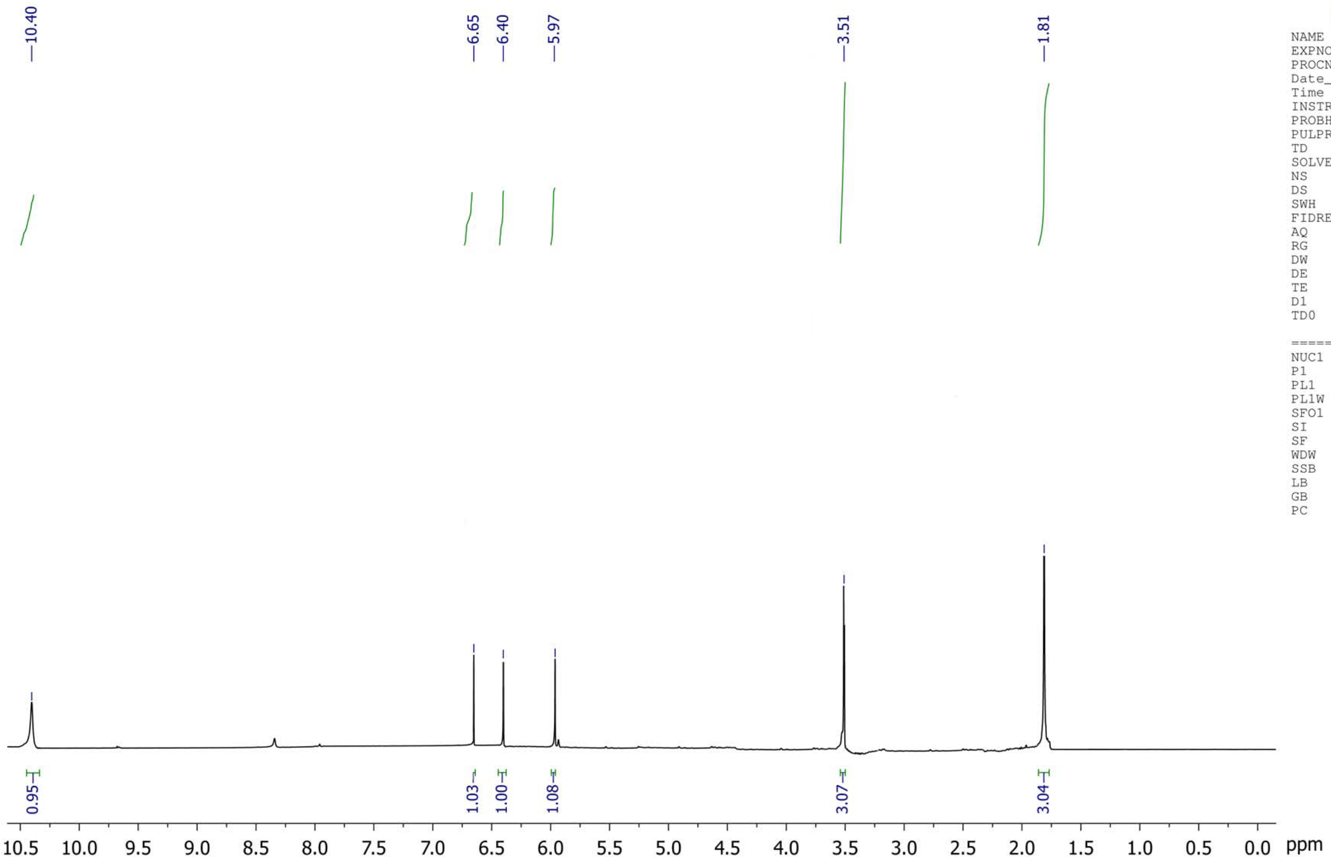

BRUKER

NAME	Yasooj UN	
EXPNO	919	
PROCNO	1	
Date_	20170217	
Time	9.42	
INSTRUM	spect	
PROBHD	5 mm PABBO BB-	
PULPROG	zg30	
TD	65536	
SOLVENT	DMSO	
NS	40	
DS	0	
SWH	8012.820	Hz
FIDRES	0.122266	Hz
AQ	4.0894966	sec
RG	40.3	
DW	62.400	usec
DE	6.50	usec
TE	293.7	K
D1	4.00000000	sec
TD0	1	

======== CHANNEL f1 ========

NUC1	1H	
P1	14.00	usec
PL1	-2.00	dB
PL1W	11.86359406	W
SFO1	400.2236020	MHz
SI	32768	
SF	400.2200000	MHz
WDW	EM	
SSB	0	
LB	0.30	Hz
GB	0	
PC	1.00	

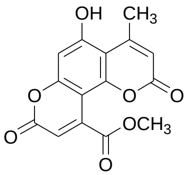
OH
CH3
O
O
O
O
OCH3
O

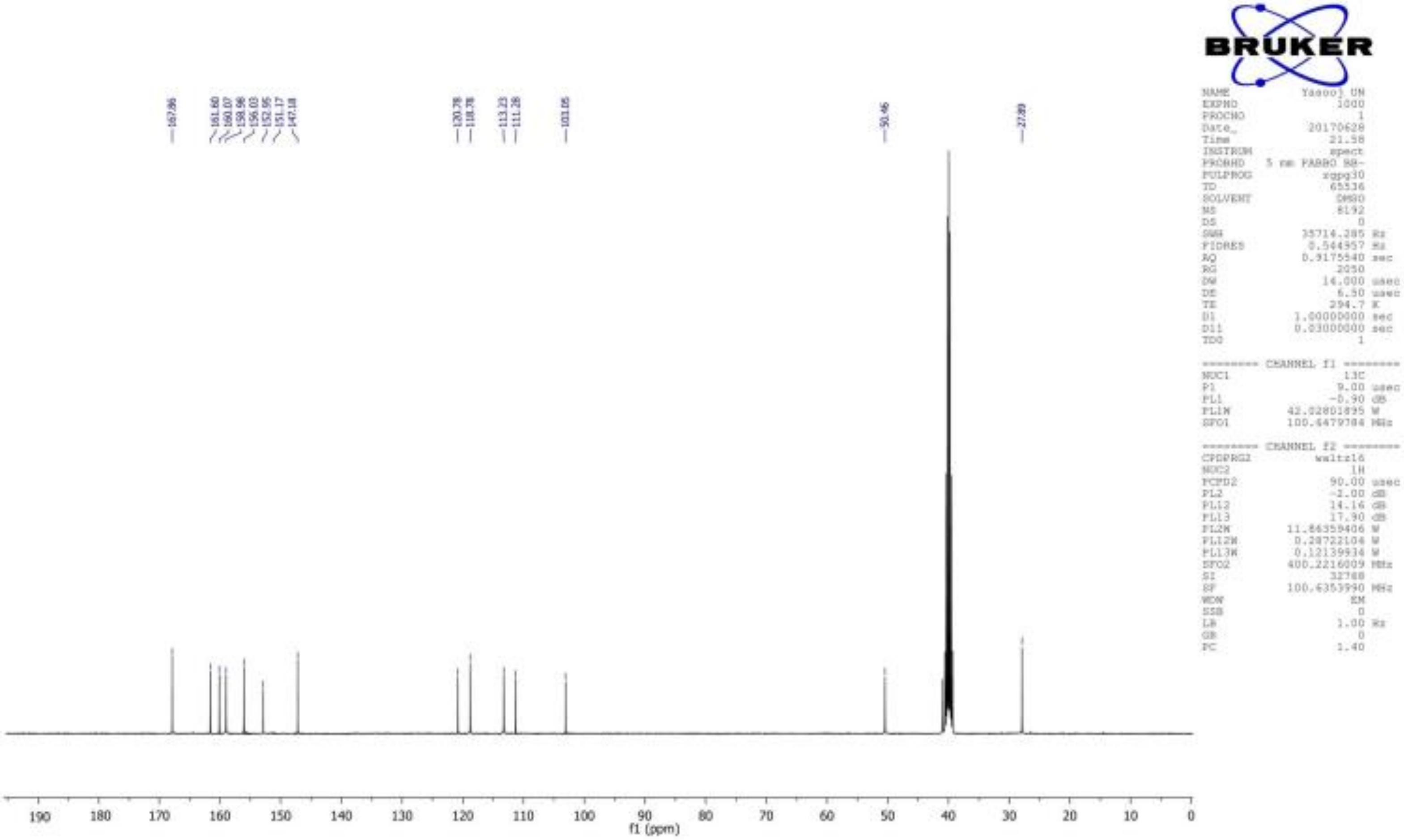
167.86
161.60
160.07
158.98
156.03
152.95
151.17
147.18
120.78
118.78
113.23
111.28
103.05
50.46
27.89
190 180 170 160 150 140 130 120 110 100 90 80 70 60 50 40 30 20 10 0
f1 (ppm)
NAME Yasooj UN
EXPNO 1000
PROCNO 1
Date_ 20170628
Time 21.58
INSTRUM spect
PROBHD 5 mm PABBO BB-
PULPROG zgpg30
TD 65536
SOLVENT DMSO
NS 8192
DS 0
SWH 35714.285 Hz
FIDRES 0.544957 Hz
AQ 0.9175540 sec
RG 2050
DW 14.000 usec
DE 6.50 usec
TE 294.7 K
D1 1.00000000 sec
D11 0.03000000 sec
TD0 1
======== CHANNEL f1 ========
NUC1 13C
P1 9.00 usec
PL1 -0.90 dB
PL1W 42.02801895 W
SFO1 100.6479784 MHz
======== CHANNEL f2 ========
CPDPRG2 waltz16
NUC2 1H
PCPD2 90.00 usec
PL2 -2.00 dB
PL12 14.16 dB
PL13 17.90 dB
PL2W 11.86359406 W
PL12W 0.28722104 W
PL13W 0.12139934 W
SFO2 400.2216009 MHz
SI 32768
SF 100.6353990 MHz
WDW EM
SSB 0
LB 1.00 Hz
GB 0
PC 1.40

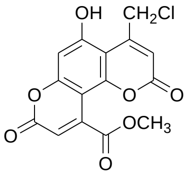

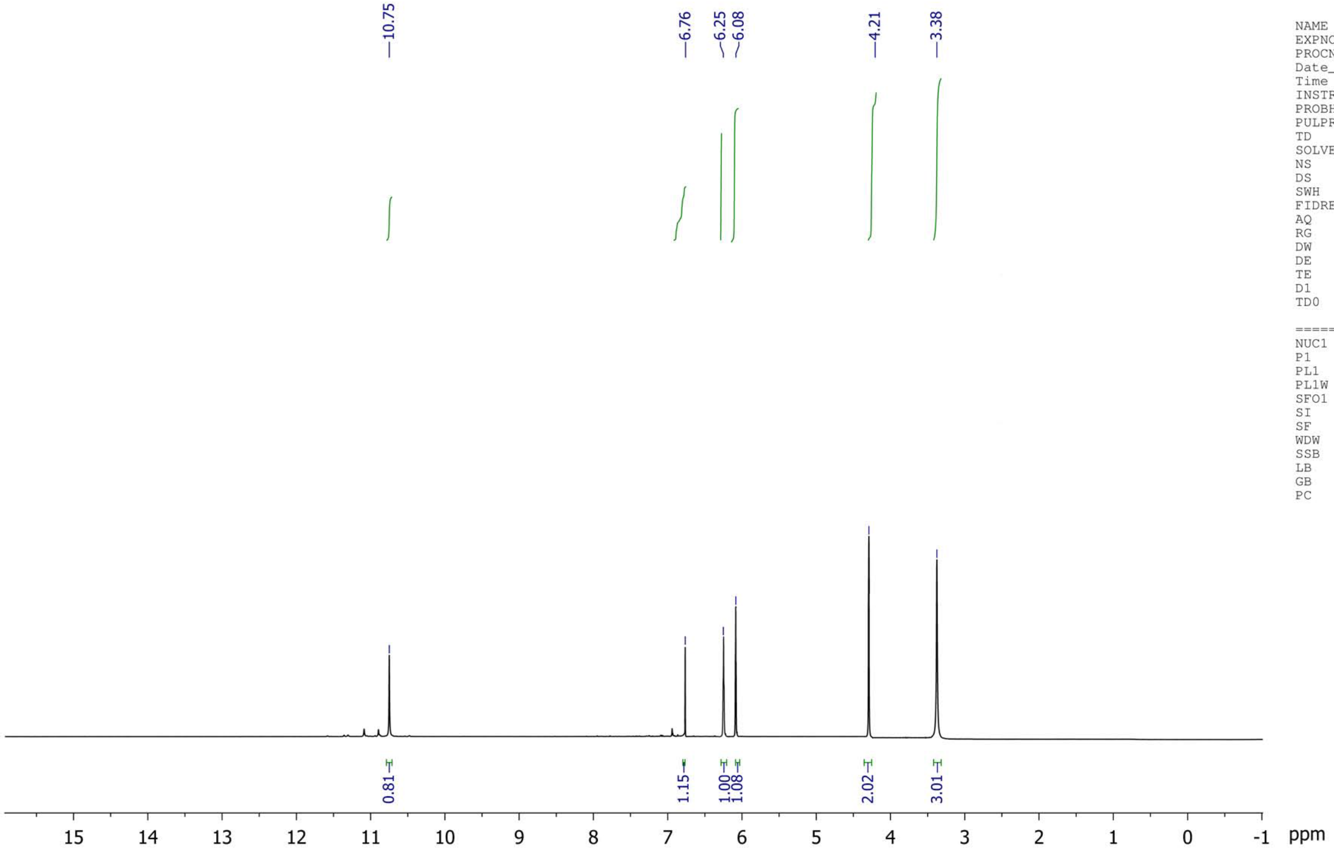

NAME	Yasooj UN
EXPNO	963
PROCNO	1
Date_	20170502
Time	11.56
INSTRUM	spect
PROBHD	5 mm PABBO BB-
PULPROG	zg30
TD	65536
SOLVENT	DMSO
NS	20
DS	0
SWH	8012.820 Hz
FIDRES	0.122266 Hz
AQ	4.0894966 sec
RG	161
DW	62.400 usec
DE	6.50 usec
TE	293.9 K
D1	4.00000000 sec
TD0	1

======== CHANNEL f1 ========

NUC1	1H
P1	14.00 usec
PL1	-2.00 dB
PL1W	11.86359406 W
SFO1	400.2236020 MHz
SI	32768
SF	400.2200000 MHz
WDW	EM
SSB	0
LB	0.30 Hz
GB	0
PC	1.00

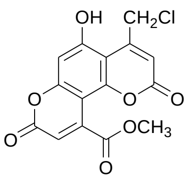
OH
CH2Cl
O
O
O
O
OCH3
O

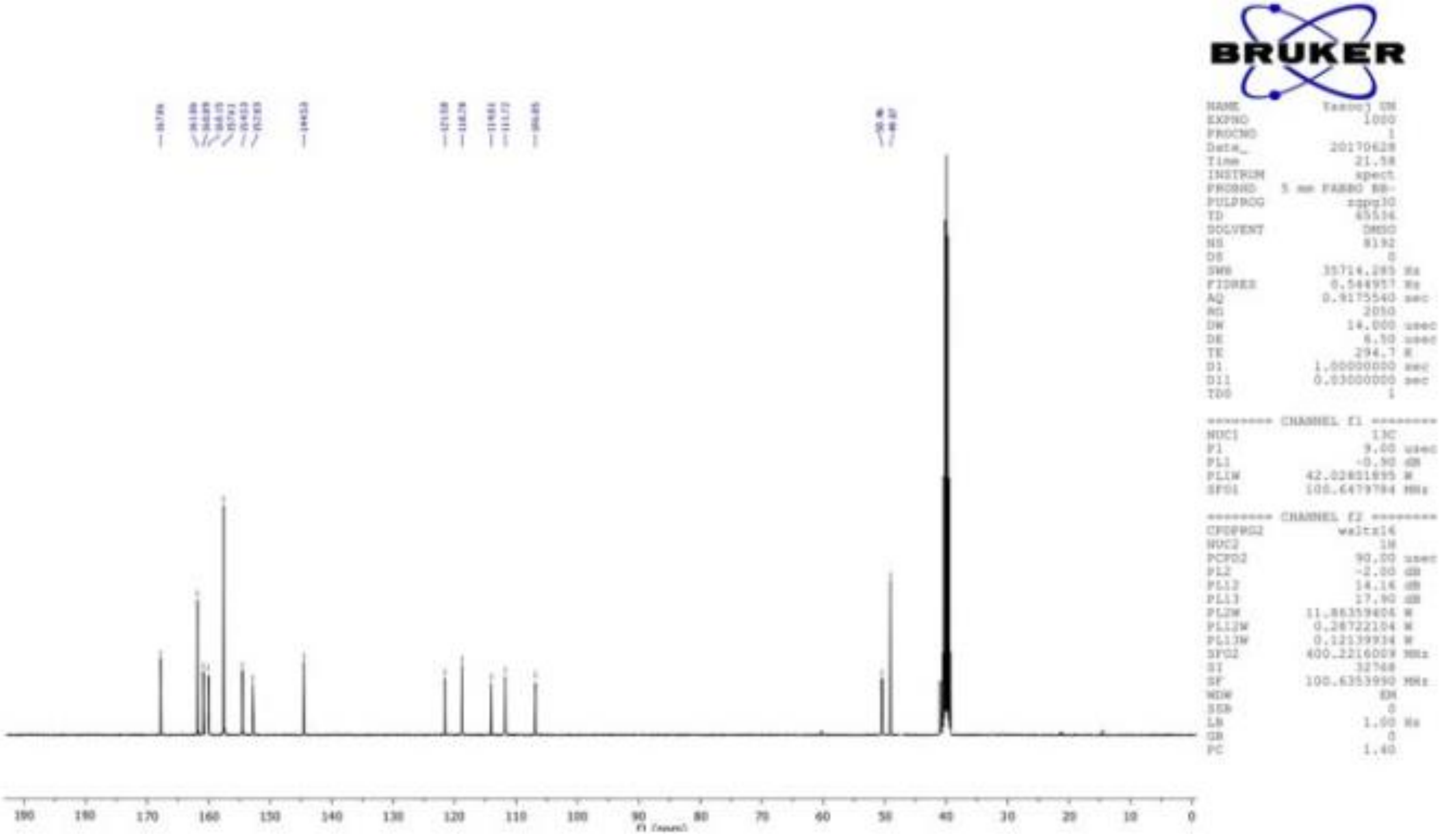
NAME Yasooj UH
EXPNO 1000
PROCNO 1
Date_ 20170628
Time 21.58
INSTRUM spect
PROBHD 5 mm PABBO BB-
PULPROG zgpg30
TD 65536
SOLVENT DMSO
NS 8192
DS 0
SWH 35714.285 Hz
FIDRES 0.544957 Hz
AQ 0.9175540 sec
RG 2050
DW 14.000 usec
DE 6.50 usec
TE 294.7 K
D1 1.00000000 sec
D11 0.03000000 sec
TD0 1
======== CHANNEL f1 ========
NUC1 13C
P1 9.00 usec
PL1 -0.90 dB
PL1W 42.02851895 W
SFO1 100.6479784 MHz
======== CHANNEL f2 ========
CPDPRG2 waltz16
NUC2 1H
PCPD2 90.00 usec
PL2 -2.00 dB
PL12 14.16 dB
PL13 17.90 dB
PL2W 11.86359406 W
PL12W 0.28722104 W
PL13W 0.12139934 W
SFO2 400.2216009 MHz
SI 32768
SF 100.6353990 MHz
WDW EM
SSB 0
LB 1.00 Hz
GB 0
PC 1.40
170
160
150
140
130
120
110
100
90
80
70
60
50
40
30
20
10
0
f1 (ppm)

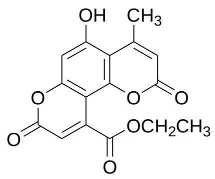

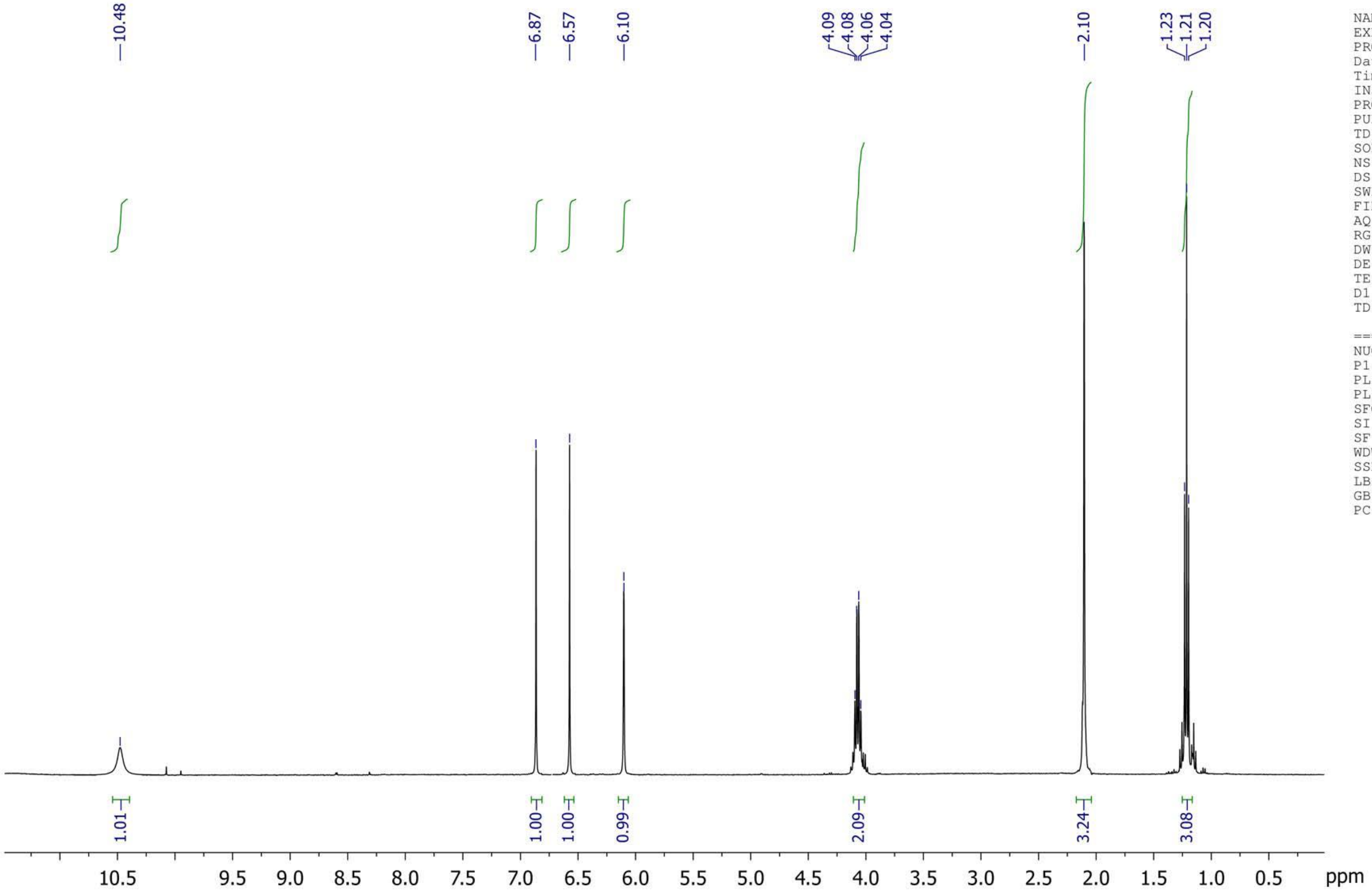

NAME	Yasooj UN
EXPNO	964
PROCNO	1
Date_	20170502
Time	12.02
INSTRUM	spect
PROBHD	5 mm PABBO BB-
PULPROG	zg30
TD	65536
SOLVENT	DMSO
NS	20
DS	0
SWH	8012.820 Hz
FIDRES	0.122266 Hz
AQ	4.0894966 sec
RG	161
DW	62.400 usec
DE	6.50 usec
TE	293.9 K
D1	4.00000000 sec
TD0	1

======== CHANNEL f1 ========

NUC1	1H
P1	14.00 usec
PL1	-2.00 dB
PL1W	11.86359406 W
SFO1	400.2236020 MHz
SI	32768
SF	400.2200000 MHz
WDW	EM
SSB	0
LB	0.30 Hz
GB	0
PC	1.00

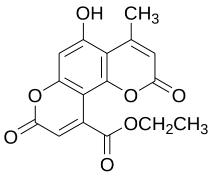

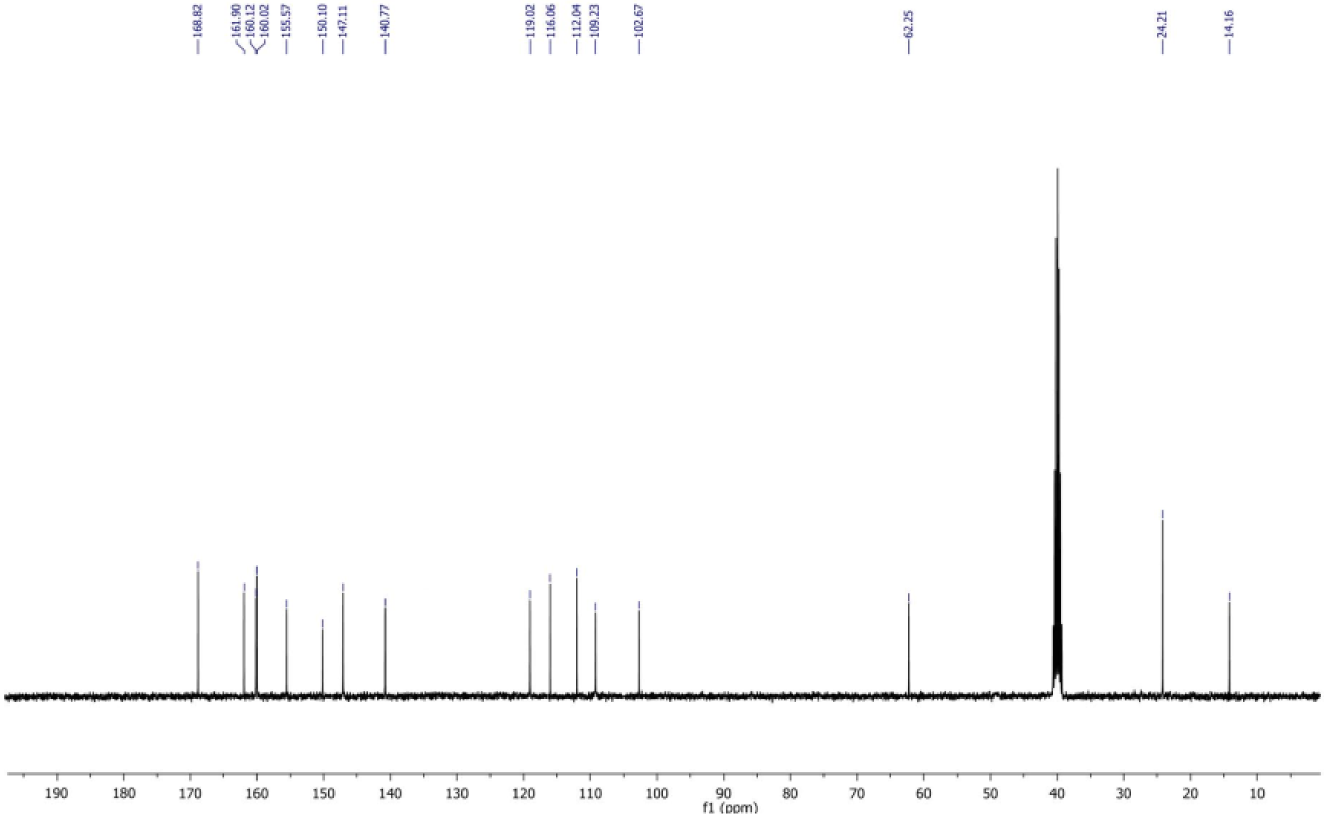

BRUKER

```
NAME          Tehran UN
EXPNO               642
PROCNO                1
Date_          20170628
Time              13.58
INSTRUM           spect
PROBHD   5 mm PABBO BB-
PULPROG          zgpg30
TD                65536
SOLVENT            DMSO
NS                  200
DS                    0
SWH           35714.285 Hz
FIDRES         0.544957 Hz
AQ            0.9175540 sec
RG                 2050
DW               14.000 usec
DE                 6.50 usec
TE                294.3 K
D1           1.00000000 sec
D11          0.03000000 sec
TD0                   1

======== CHANNEL f1 ========
NUC1                13C
P1                 9.00 usec
PL1               -0.90 dB
PL1W        42.02801895 W
SFO1        100.6479784 MHz

======== CHANNEL f2 ========
CPDPRG2         waltz16
NUC2                 1H
PCPD2             90.00 usec
PL2               -2.00 dB
PL12              14.48 dB
PL13              17.90 dB
PL2W        11.86359406 W
PL12W        0.26681873 W
PL13W        0.12139934 W
SFO2        400.2216009 MHz
SI                32768
SF          100.6353990 MHz
WDW                  EM
SSB                   0
LB                 1.00 Hz
GB                    0
PC                 1.40
```

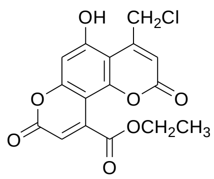
OH
CH2Cl
O
O
O
O
OCH2CH3
O

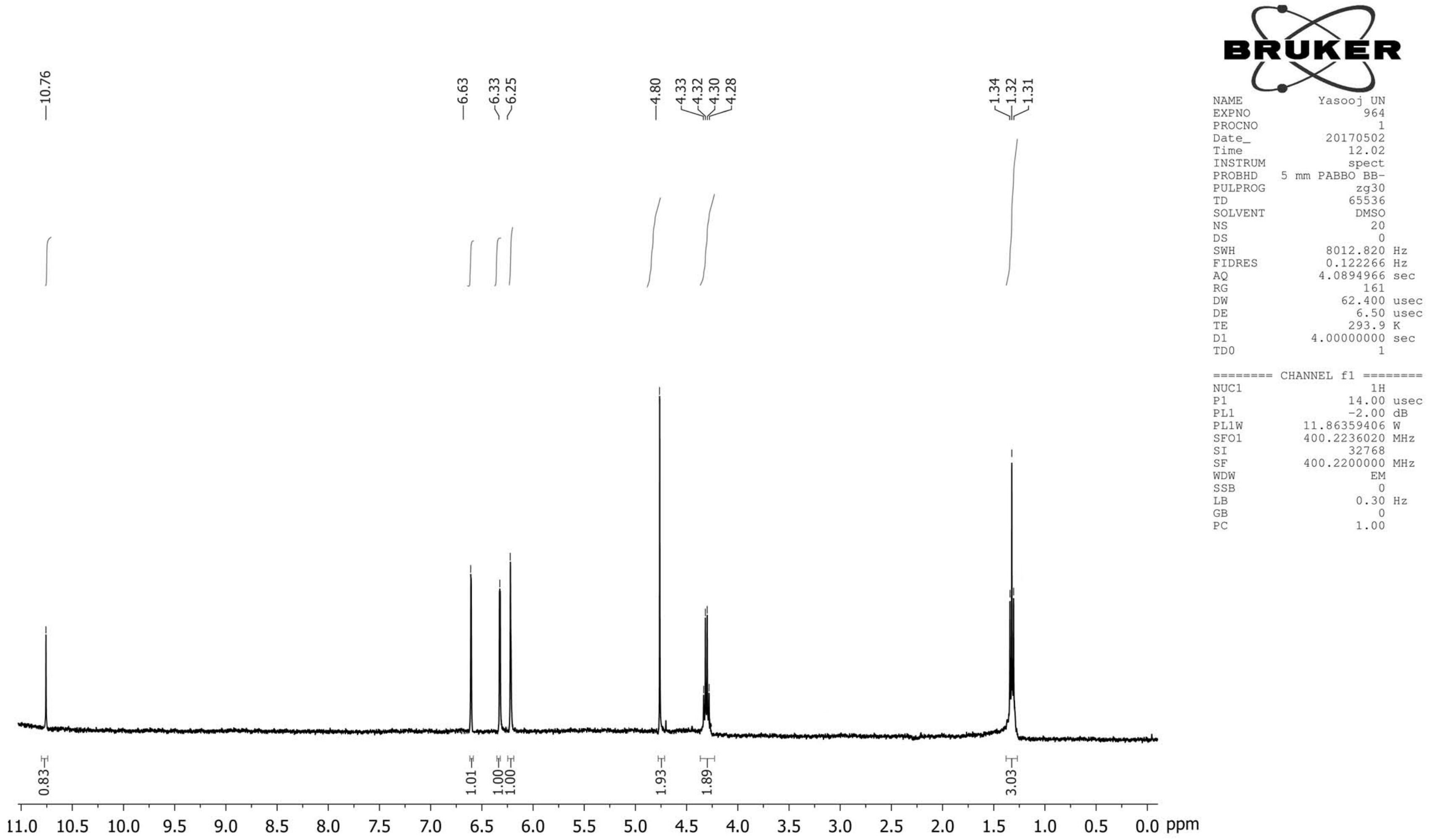
10.76
6.63
6.33
6.25
4.80
4.33
4.32
4.30
4.28
1.34
1.32
1.31
0.83
1.01
1.00
1.00
1.93
1.89
3.03
11.0 10.5 10.0 9.5 9.0 8.5 8.0 7.5 7.0 6.5 6.0 5.5 5.0 4.5 4.0 3.5 3.0 2.5 2.0 1.5 1.0 0.5 0.0 ppm
NAME Yasooj UN
EXPNO 964
PROCNO 1
Date_ 20170502
Time 12.02
INSTRUM spect
PROBHD 5 mm PABBO BB-
PULPROG zg30
TD 65536
SOLVENT DMSO
NS 20
DS 0
SWH 8012.820 Hz
FIDRES 0.122266 Hz
AQ 4.0894966 sec
RG 161
DW 62.400 usec
DE 6.50 usec
TE 293.9 K
D1 4.00000000 sec
TD0 1
======== CHANNEL f1 ========
NUC1 1H
P1 14.00 usec
PL1 -2.00 dB
PL1W 11.86359406 W
SFO1 400.2236020 MHz
SI 32768
SF 400.2200000 MHz
WDW EM
SSB 0
LB 0.30 Hz
GB 0
PC 1.00

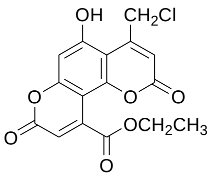
OH
CH2Cl
O
O
O
O
OCH2CH3
O

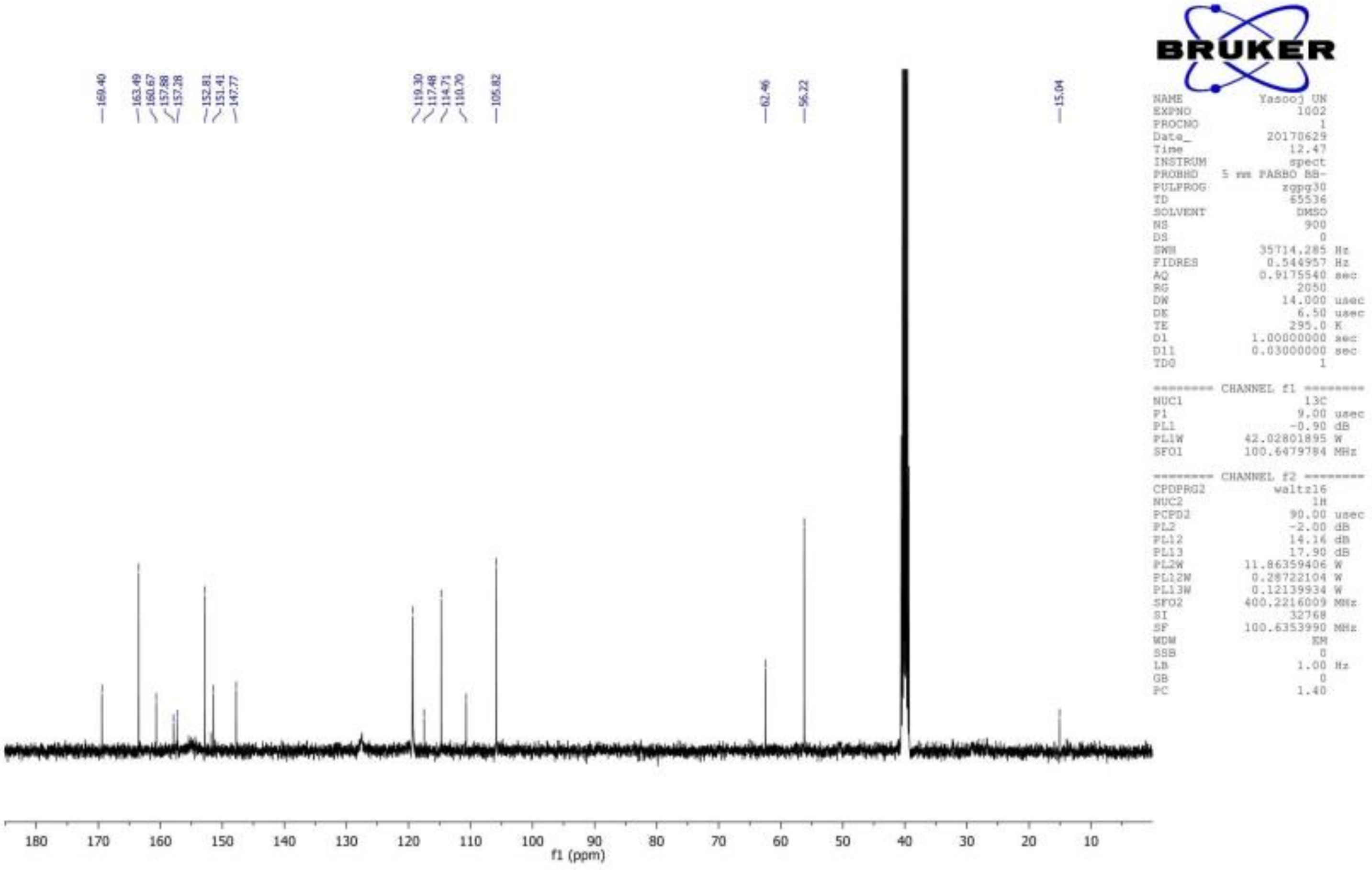
169.40
163.49
160.67
157.88
157.28
152.81
151.41
147.77
119.30
117.48
114.71
110.70
105.82
62.46
56.22
15.04
180 170 160 150 140 130 120 110 100 90 80 70 60 50 40 30 20 10
f1 (ppm)
BRUKER
NAME Yasooj UN
EXPNO 1002
PROCNO 1
Date_ 20170629
Time 12.47
INSTRUM spect
PROBHD 5 mm PABBO BB-
PULPROG zgpg30
TD 65536
SOLVENT DMSO
NS 900
DS 0
SWH 35714.285 Hz
FIDRES 0.544957 Hz
AQ 0.9175540 sec
RG 2050
DW 14.000 usec
DE 6.50 usec
TE 295.0 K
D1 1.00000000 sec
D11 0.03000000 sec
TD0 1
======== CHANNEL f1 ========
NUC1 13C
P1 9.00 usec
PL1 -0.90 dB
PL1W 42.02801895 W
SFO1 100.6479784 MHz
======== CHANNEL f2 ========
CPDPRG2 waltz16
NUC2 1H
PCPD2 90.00 usec
PL2 -2.00 dB
PL12 14.16 dB
PL13 17.90 dB
PL2W 11.86359406 W
PL12W 0.28722104 W
PL13W 0.12139934 W
SFO2 400.2216009 MHz
SI 32768
SF 100.6353990 MHz
WDW EM
SSB 0
LB 1.00 Hz
GB 0
PC 1.40

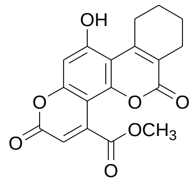

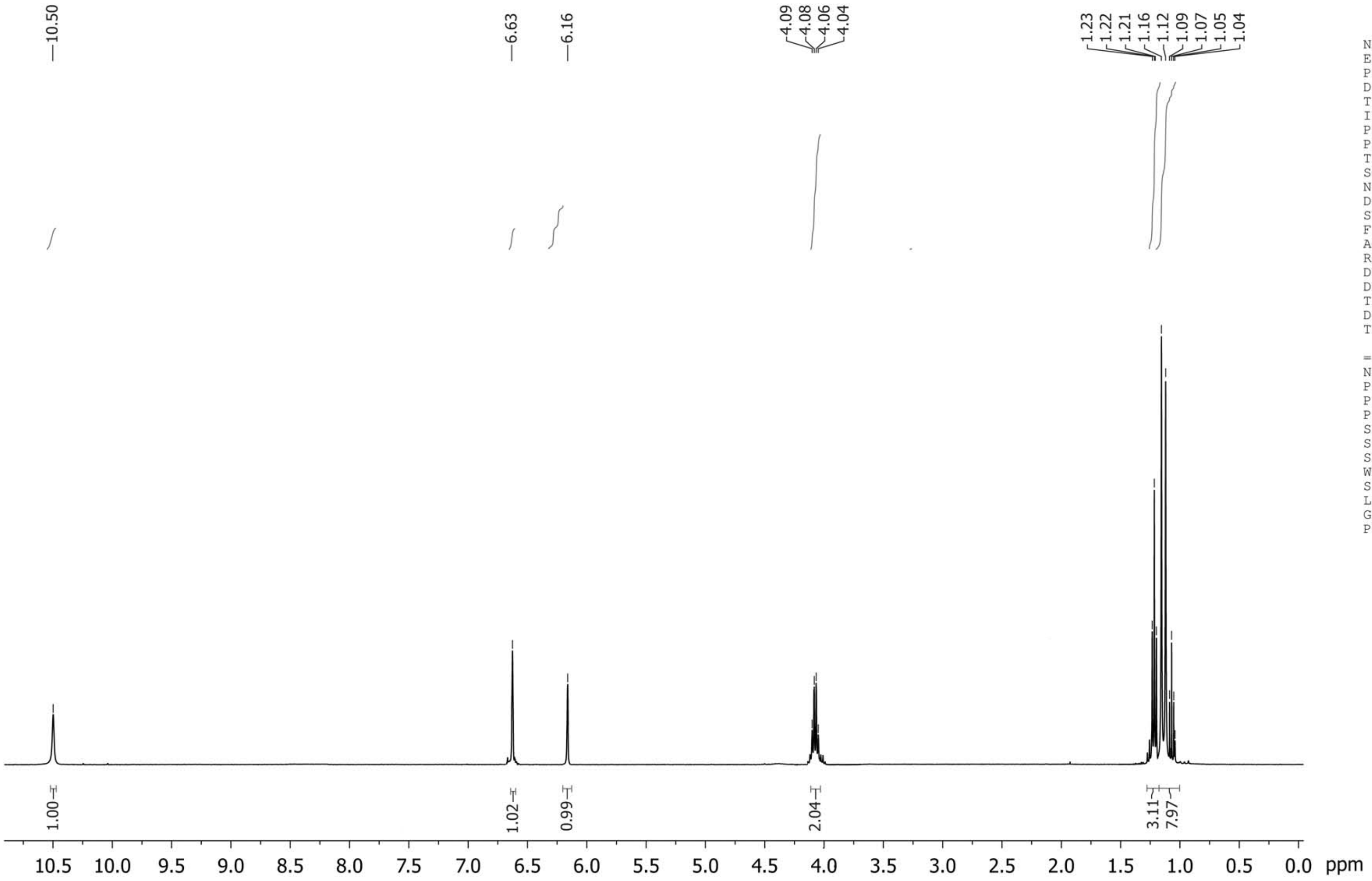


```
NAME        Yasooj UN
EXPNO             891
PROCNO              1
Date_        20161226
Time            17.07
INSTRUM         spect
PROBHD   5 mm PABBO BB-
PULPROG          zg30
TD              65536
SOLVENT          DMSO
NS                 20
DS                  0
SWH          8012.820 Hz
FIDRES       0.122266 Hz
AQ          4.0894966 sec
RG                203
DW             62.400 usec
DE               6.50 usec
TE              293.8 K
D1         4.00000000 sec
TD0                 1

======== CHANNEL f1 ========
NUC1               1H
P1              14.00 usec
PL1             -2.00 dB
PL1W      11.86359406 W
SFO1      400.2236020 MHz
SI              32768
SF        400.2200000 MHz
WDW                EM
SSB                 0
LB               0.30 Hz
GB                  0
PC               1.00
```

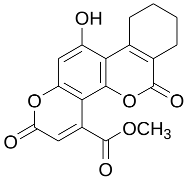
OH
O
O
O
O
OCH3
O

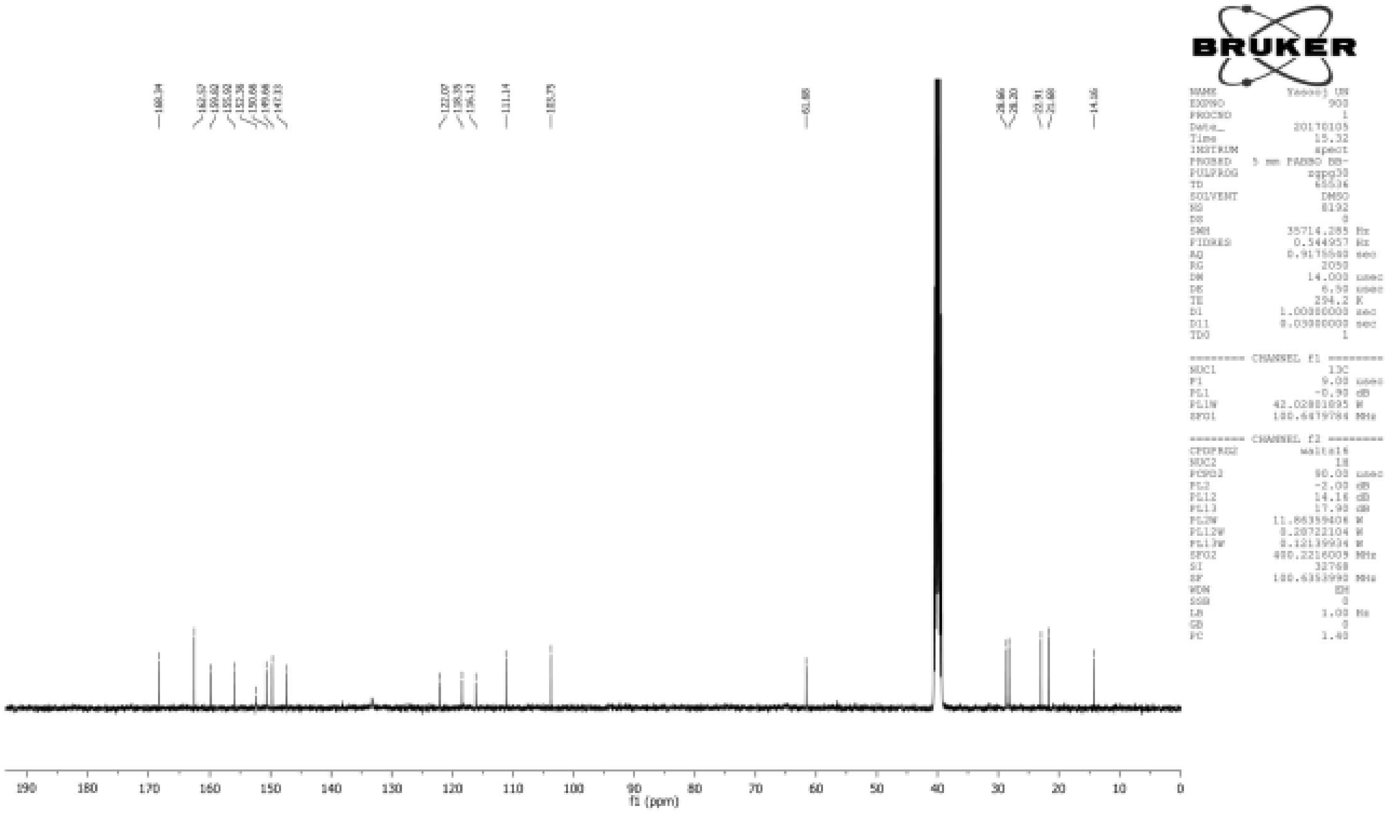
190
180
170
160
150
140
130
120
110
100
90
80
70
60
50
40
30
20
10
0
f1 (ppm)

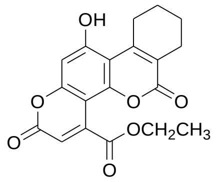

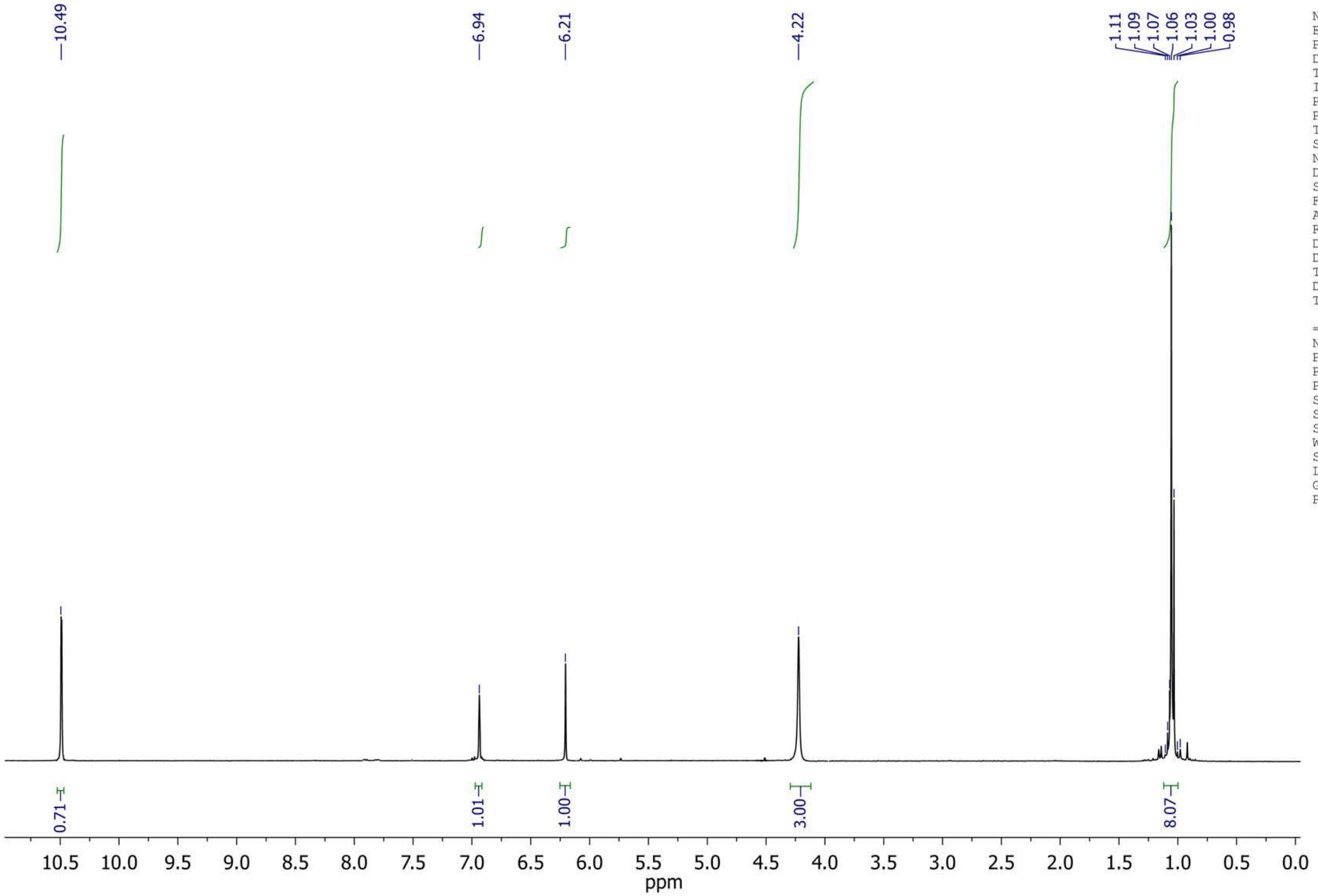

NAME	Yasooj UN	
EXPNO	892	
PROCNO	1	
Date_	20161226	
Time	17.15	
INSTRUM	spect	
PROBHD	5 mm PABBO BB-	
PULPROG	zg30	
TD	65536	
SOLVENT	DMSO	
NS	20	
DS	0	
SWH	8012.820	Hz
FIDRES	0.122266	Hz
AQ	4.0894966	sec
RG	203	
DW	62.400	usec
DE	6.50	usec
TE	293.8	K
D1	4.00000000	sec
TD0	1	

======== CHANNEL f1 ========

NUC1	1H	
P1	14.00	usec
PL1	-2.00	dB
PL1W	11.86359406	W
SFO1	400.2236020	MHz
SI	32768	
SF	400.2200000	MHz
WDW	EM	
SSB	0	
LB	0.30	Hz
GB	0	
PC	1.00	

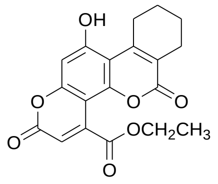
OH
O
O
O
O
OCH2CH3
O

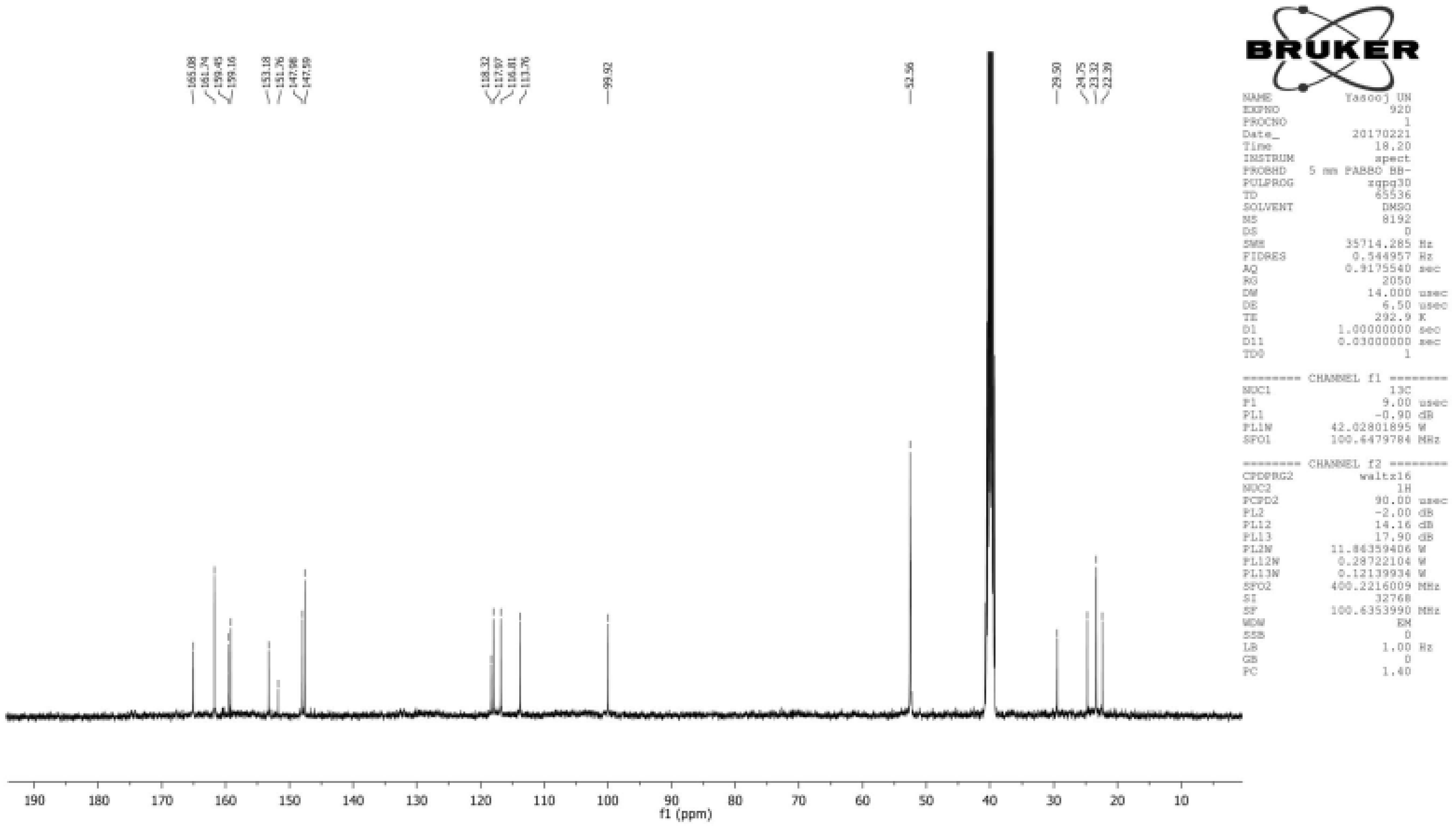
165.08
161.74
159.45
159.16
153.18
151.76
147.98
147.59
118.32
117.97
116.81
113.76
99.92
52.56
29.50
24.75
23.32
22.39
190 180 170 160 150 140 130 120 110 100 90 80 70 60 50 40 30 20 10
f1 (ppm)
NAME Yasooj UN
EXPNO 920
PROCNO 1
Date_ 20170221
Time 18.20
INSTRUM spect
PROBHD 5 mm PABBO BB-
PULPROG zgpg30
TD 65536
SOLVENT DMSO
NS 8192
DS 0
SWH 35714.285 Hz
FIDRES 0.544957 Hz
AQ 0.9175540 sec
RG 2050
DW 14.000 usec
DE 6.50 usec
TE 292.9 K
D1 1.00000000 sec
D11 0.03000000 sec
TD0 1
======== CHANNEL f1 ========
NUC1 13C
P1 9.00 usec
PL1 -0.90 dB
PL1W 42.02801895 W
SFO1 100.6479784 MHz
======== CHANNEL f2 ========
CPDPRG2 waltz16
NUC2 1H
PCPD2 90.00 usec
PL2 -2.00 dB
PL12 14.16 dB
PL13 17.90 dB
PL2W 11.86359406 W
PL12W 0.28722104 W
PL13W 0.12139934 W
SFO2 400.2216009 MHz
SI 32768
SF 100.6353990 MHz
WDW EM
SSB 0
LB 1.00 Hz
GB 0
PC 1.40

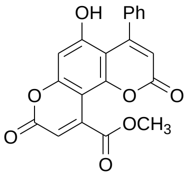

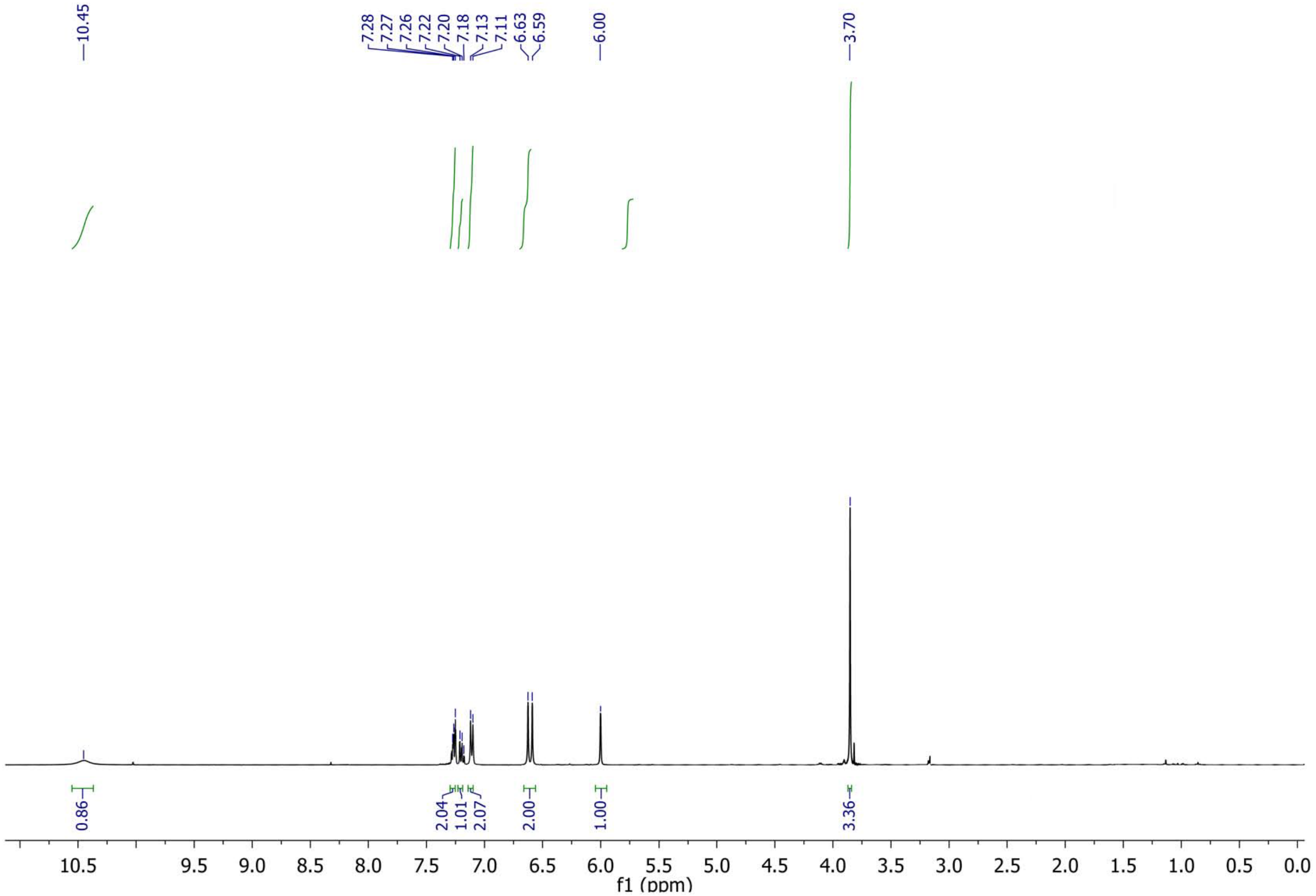

NAME	Yasooj UN
EXPNO	898
PROCNO	1
Date_	20171226
Time	17.15
INSTRUM	spect
PROBHD	5 mm PABBO BB-
PULPROG	zg30
TD	65536
SOLVENT	DMSO
NS	20
DS	0
SWH	8012.820 Hz
FIDRES	0.122266 Hz
AQ	4.0894966 sec
RG	203
DW	62.400 usec
DE	6.50 usec
TE	293.8 K
D1	4.00000000 sec
TD0	1

======== CHANNEL f1 ========

NUC1	1H
P1	14.00 usec
PL1	-2.00 dB
PL1W	11.86359406 W
SFO1	400.2236020 MHz
SI	32768
SF	400.2200000 MHz
WDW	EM
SSB	0
LB	0.30 Hz
GB	0
PC	1.00

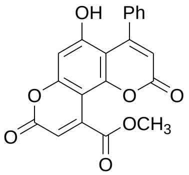
OH
Ph
O
O
O
O
OCH3
O

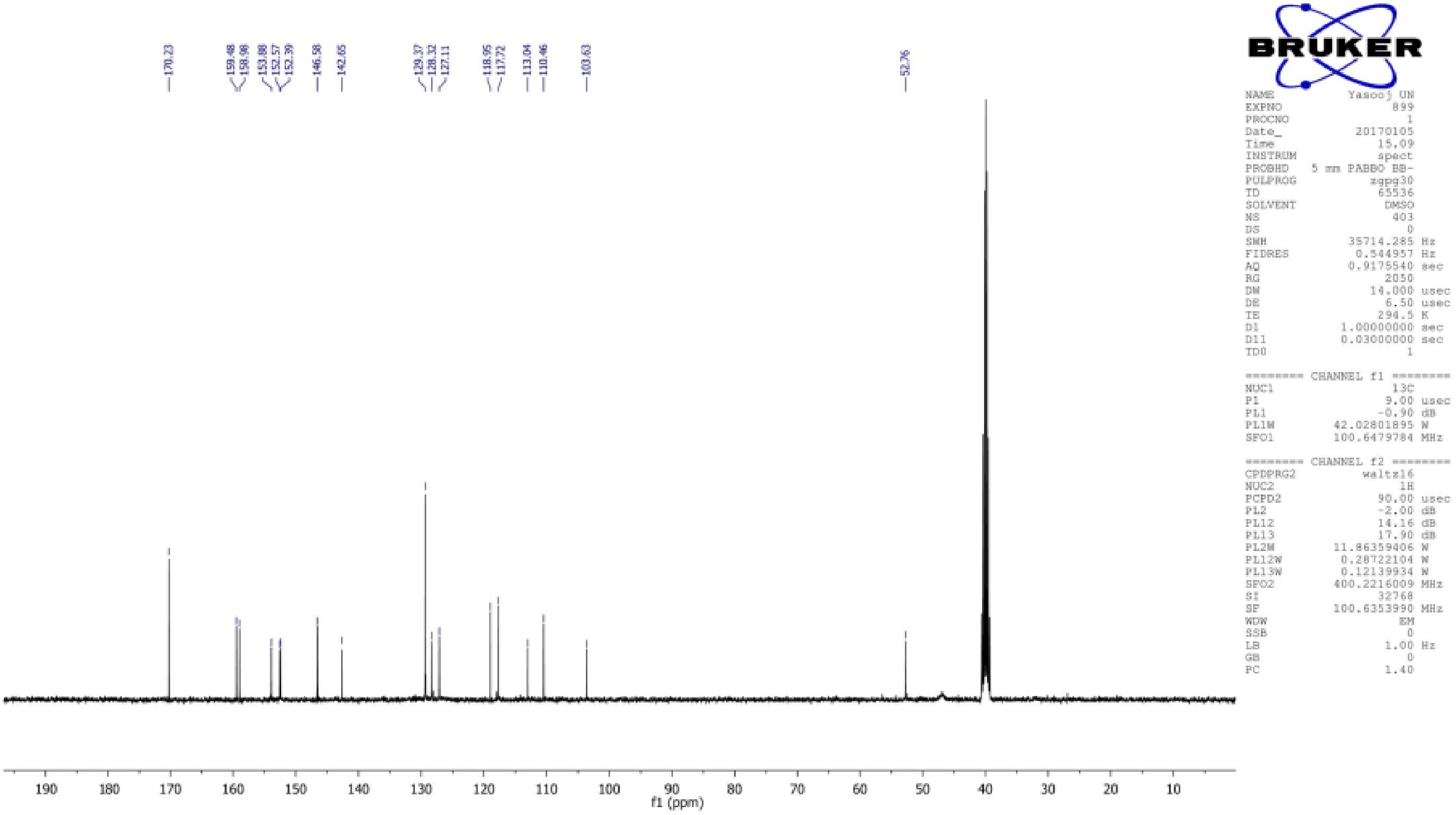
170.23
159.48
158.98
153.88
152.57
152.39
146.58
142.65
129.37
128.32
127.11
118.95
117.72
113.04
110.46
103.63
52.76
BRUKER
NAME Yasooj UN
EXPNO 899
PROCNO 1
Date_ 20170105
Time 15.09
INSTRUM spect
PROBHD 5 mm PABBO BB-
PULPROG zgpg30
TD 65536
SOLVENT DMSO
NS 403
DS 0
SWH 35714.285 Hz
FIDRES 0.544957 Hz
AQ 0.9175540 sec
RG 2050
DW 14.000 usec
DE 6.50 usec
TE 294.5 K
D1 1.00000000 sec
D11 0.03000000 sec
TD0 1
======== CHANNEL f1 ========
NUC1 13C
P1 9.00 usec
PL1 -0.90 dB
PL1W 42.02801895 W
SFO1 100.6479784 MHz
======== CHANNEL f2 ========
CPDPRG2 waltz16
NUC2 1H
PCPD2 90.00 usec
PL2 -2.00 dB
PL12 14.16 dB
PL13 17.90 dB
PL2W 11.86359406 W
PL12W 0.28722104 W
PL13W 0.12139934 W
SFO2 400.2216009 MHz
SI 32768
SF 100.6353990 MHz
WDW EM
SSB 0
LB 1.00 Hz
GB 0
PC 1.40
190
180
170
160
150
140
130
120
110
100
90
80
70
60
50
40
30
20
10
f1 (ppm)
